# Epigenetic modulation of stromal cell states underpins pathological tissue niches in Crohn’s disease

**DOI:** 10.1038/s41590-026-02617-0

**Published:** 2026-08-12

**Authors:** Simon Koplev, Osheen Sharma, Simon Woelfel, Ni Huang, Mathilde Pohin, Adrian Feile, Maria Warschinke, Mikel Rezola Artero, Sharujan Suthakaran, Ioannis Sarropoulos, J. Patrick Pett, Julia Nyman, Tom Thomas, Duy Pham, Bin Li, Moustafa Attar, Julia Pakpoor, Kate Milosevic-Hutton, Alistair Easton, Matt Butler, James Dunford, Martin Philpott, Mark Coles, Christopher D. Buckley, Calliope Dendrou, Khalid Shamiyah, Lorenz Kretschmer, Lisa Dratva, Fadi Issa, Joanna Hester, Jens Rittscher, Alissa Walsh, Simon P. Travis, Fränze Progatzky, Lukas W. Unger, Mark Bignell, Katherine Baker, Bruce George, Hussein Al-Mossawi, Paul Klenerman, Alexander S. Mosig, Udo Oppermann, Sarah A. Teichmann, Fiona M. Powrie, Matthias Friedrich

**Affiliations:** 1 Cambridge Stem Cell Institute, Jeffrey Cheah Biomedical Centre, Cambridge Biomedical Campus, Cambridge, UK; 2 Department of Gene Technology, KTH Royal Institute of Technology, Science for Life Laboratory, Stockholm, Sweden; 3 Translational Gastroenterology and Liver Unit, University of Oxford, Oxford, UK; 4 Wellcome Sanger Institute, Cambridge, UK; 5 ImmunoConcept University of Bordeaux, Immunology and Immunogenetic Unit University Hospital of Bordeaux, Bordeaux, France; 6 Kennedy Institute of Rheumatology, University of Oxford, Oxford, UK; 7 Institute of Biochemistry II, Jena University Hospital, Jena, Germany; 8 Big Data Institute/Li Ka Shing Centre for Health Information and Discovery, University of Oxford, Oxford, UK; 9 NIHR Oxford Biomedical Research Centre Oxford, Oxford, UK; 10 Department of Oncology, University of Oxford, Oxford, UK; 11 UCB Pharma, Slough, UK; 12 Botnar Institute, University of Oxford, Oxford, UK; 13 Oxford Translational Myeloma Centre, Botnar Research Centre, University of Oxford, Oxford, UK; 14 Nuffield Department of Orthopedics, Rheumatology and Musculoskeletal Sciences, University of Oxford, Oxford, UK; 15 Nuffield Department of Surgical Sciences, University of Oxford, Oxford, UK; 16 CAMS Oxford Institute, Chinese Academy of Medical Sciences & Peking Union Medical College, University of Oxford, Oxford, UK; 17 Institute of Biomedical Engineering (IBME), Department of Engineering Science, University of Oxford, Oxford, UK; 18 Ludwig Institute for Cancer Research (Oxford Branch), University of Oxford, Oxford, UK; 19 Department of Colorectal Surgery, Oxford University Hospitals, Oxford, UK; 20 Peter Medawar Building, South Parks Rd, University of Oxford, Oxford, UK; 21 Pandemic Sciences Institute, University of Oxford, Oxford, UK

**Keywords:** Crohn's disease, Chronic inflammation

## Abstract

Stromal cell states are altered in active Crohn’s disease (CD), but their origin and phenotypic stability are unknown. Using single-cell spatial transcriptomics, RNA sequencing and ATAC sequencing of ~2,500,000 cells, we map 18 distinct stromal cell states within their cellular and cytokine signaling environments in human full-thickness CD bowel. Inflammatory fibroblasts (IFs) reside in immune cell-rich mucosal ulcers and are induced through combinatorial cytokine exposure suppressing submucosal universal fibroblast programs. The IF state is stabilized through transcription factor (TF) activity of GLI3, TWIST1, ETV4, PRDM1 and RELB, and does not spontaneously revert; however, histone deacetylase inhibition destabilizes the IF state, preventing IF secretome-induced epithelial transmigration and activation of neutrophils. The IF open chromatin configuration is distinct from that of fibrotic contractile stroma, which populate adjoining immune-depleted submucosal fibrotic niches. These findings show that microenvironments in pathological tissue niches shape open chromatin configuration of stromal states that are amenable to modulation by epigenetic modifiers.

## Abstract (web summary)

The authors provide a multiomic single-cell and spatial atlas of Crohn’s disease, focusing on the crosstalk between immune and stromal cells and heterogeneity around mucosal lesions.

## Main

Crohn’s disease (CD) is a heterogeneous chronic relapsing disease of the gastrointestinal tract involving inflammation and fibrosis, which can cause obstruction (stenosis) of the bowel requiring surgery^1,2^. Inflammation and fibrosis each contribute to the pathological thickening of the bowel wall, through the infiltration of high numbers of immune cells and the excessive deposition of extracellular matrix, respectively^3^. Rates of surgery in CD continue to be high despite the introduction of advanced biologic and small-molecule inhibitor therapy^4–10^, highlighting the urgent need to discover alternative avenues for medical therapy in CD.

Existing evidence links particular subsets of stromal cells to difficult-to-treat inflammatory bowel disease, including CD^11–22^. One such state is the inflammatory fibroblast (IF), a stromal cell population that we have shown to be responsive to IL-1 signaling and to reside in areas of mucosal ulceration with prominent neutrophil infiltration^11^. IFs are characterized by the expression of a range of proinflammatory molecules such as neutrophil-attracting CXC-type chemokines (CXCL5 and CXCL6), cytokines (IL-11), fibroblast activation markers podoplanin (PDPN) and fibroblast activation protein (FAP), extracellular matrix components (collagens and fibronectins) and matrix remodeling enzymes (matrix metalloproteinases (MMPs) and ADAMs). The presence of IFs is a hallmark of patients with inflammatory bowel disease (IBD) who are nonresponsive to immunosuppressive therapies such as corticosteroids, anti-tumor necrosis factor (TNF) and anti-integrin agents^11,16^. Fibroblast states that share transcriptional markers with IF have also been specifically associated with fibrostenotic CD. For example, MMP^+^WNT5A^+^CDH11^+^ (ref. ^23^), IL-11^+^ (refs. ^18,22^) and KI67^+^FAP^+^TWIST1^+^ (refs. ^21,24,25^) fibroblasts are proportionally increased in tissues and fistulas isolated from patients with fibrostenotic CD. Whether these represent ulcer-associated IF that co-present with the indication of fibrostenosis, or are distinct populations specifically found in fibrotic tissue niches is unclear. Furthermore, we lack a molecular understanding of how these pathological tissue niches shape CD-associated stromal cell states and whether these cellular states are phenotypically stable.

In this study, we provide high-resolution spatial profiling (Xenium single-cell spatial transcriptomics), mapping stromal cell states within key pathological tissue niches in CD. We reveal that spatially separated but adjacent inflamed and fibrotic niches are occupied by distinct stromal cell states: IFs localize to immune-dense mucosal ulcers, whereas contractile stroma (‘CS’; fibrotic muscle and myofibroblasts) predominates in areas of immune-depleted submucosal fibrosis. Profiling by single-cell ATAC-seq highlights characteristic and distinct open chromatin configurations of IFs and CS. Generating a stromal cytokine response atlas, we reveal that IF, unlike CS, reside in areas of high signaling activity of multiple cytokines. Consistent with this, we show that combinatorial cytokine exposure in vitro generates a stable IF state that does not revert spontaneously or upon cytokine blockade. By contrast, exposure to different epigenetic modifier compounds either destabilizes IFs, or shifts them toward contractile CS states.

These findings resolve the heterogeneity of inflammatory and fibrotic tissue niches in CD, which each harbor unique stromal cell states with distinct transcriptional and epigenetic configurations. Transition to the ulcer-associated IF state can be induced by combinatorial cytokine cues altering epigenetic configuration, which can be modulated by epigenetic modifier compounds.

## Results

### Stromal cell states in inflamed and fibrotic CD tissue niches

**Fig. 1:**
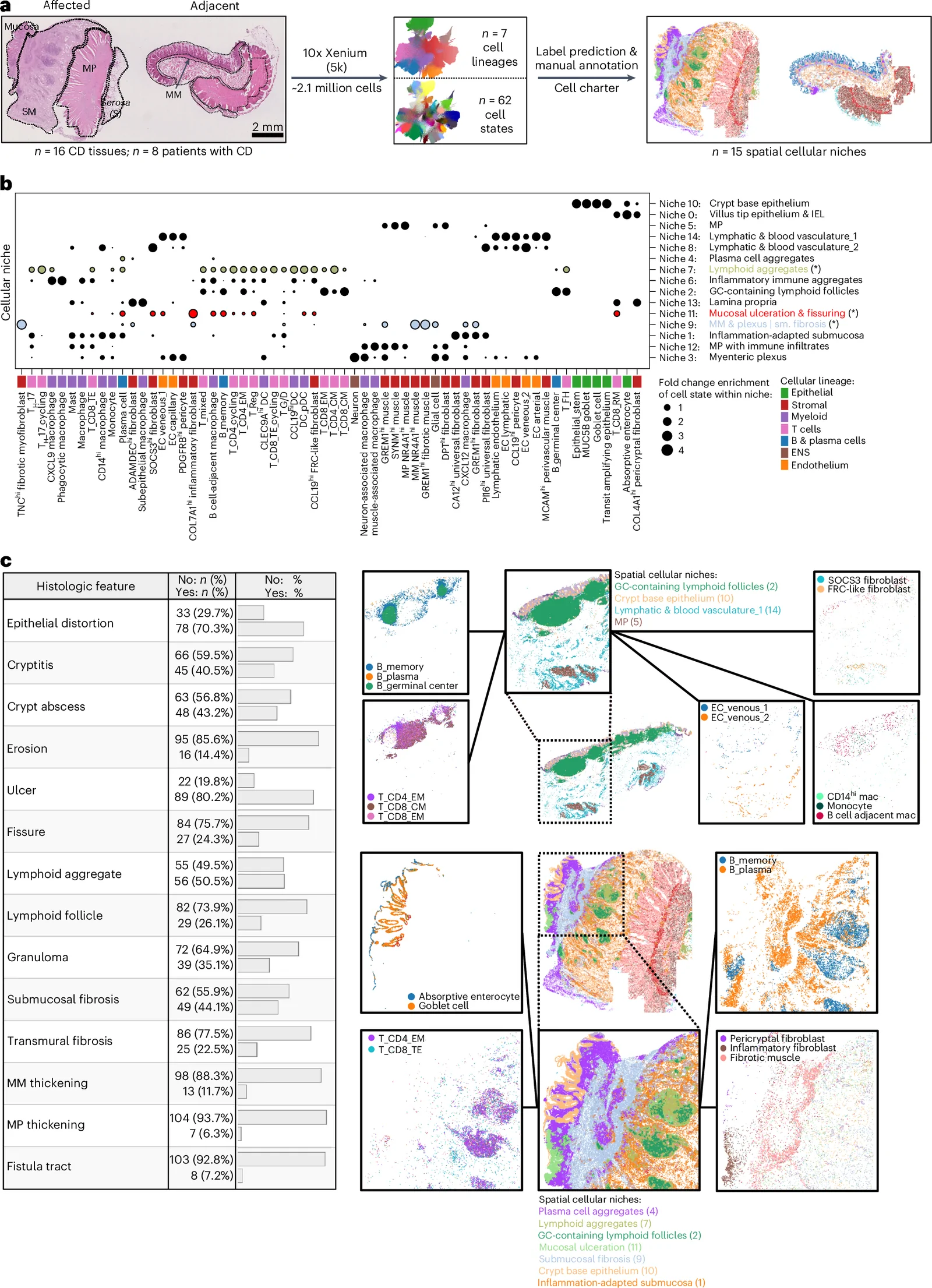
Stromal cell states in inflamed and fibrotic Crohn’s disease tissue niches. **a**, Schematic workflow of spatial transcriptomic profiling using 10x Xenium Human Prime 5,000-plex panel. MP, muscularis propria; MM, muscularis mucosae; **b**, Composition of cellular niches, showing significant relative fold enrichment of cellular states within each spatial cellular niche derived from CellCharter analysis. * indicates niches that are significantly increased in relative abundance in affected CD tissue (Extended Data Fig. 1a). **c**, Summary of the quantification of histopathologist scoring (present, yes)/absent, no) of established histopathologic features in *n* = 111 CD surgical specimens (left). The images on the right visualize examples of detected cellular niches (CellCharter) reflecting these histopathologic features. FRC, fibroblastic reticular cell; IEL, intraepithelial lymphocyte; Th, T helper; EC, endothelial cell; MP, muscularis propria; EM, effector memory; TE, terminal effector; CM, central memory.

**Extended Data Fig. 1:**
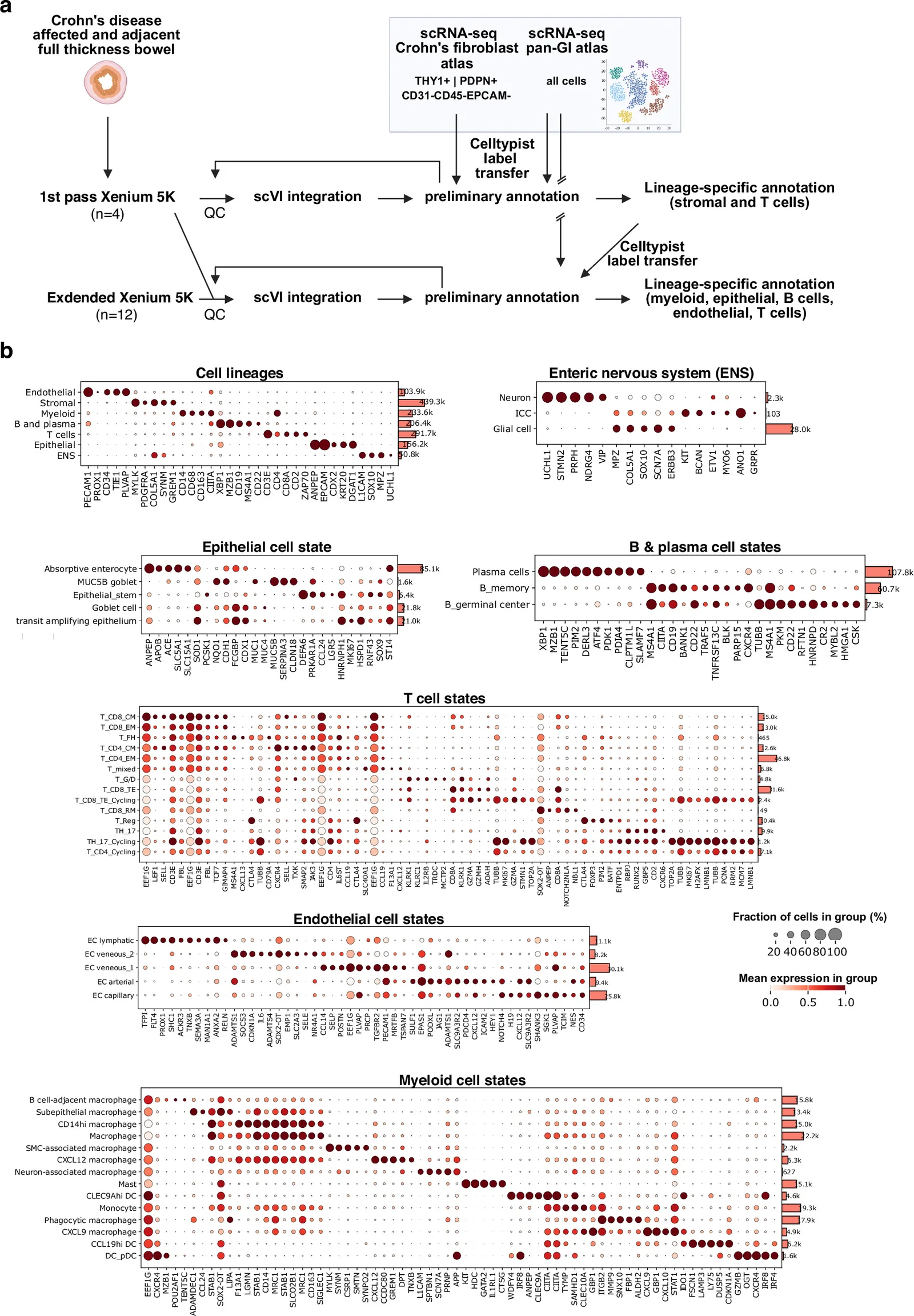
10X Xenium profiling, analysis and annotations. **a**, Schematic workflow summarizing the analysis strategy and dataset integration for annotating the full Xenium 5 K datasets comprising CD patient tissues 1-16. **b**, Dotplots of top markers for each detected cell lineage, and cell states per each cell lineage and cell state within cellular lineages. Barplots indicate the total number of cells detected per each cluster.

**Extended Data Fig. 2:**
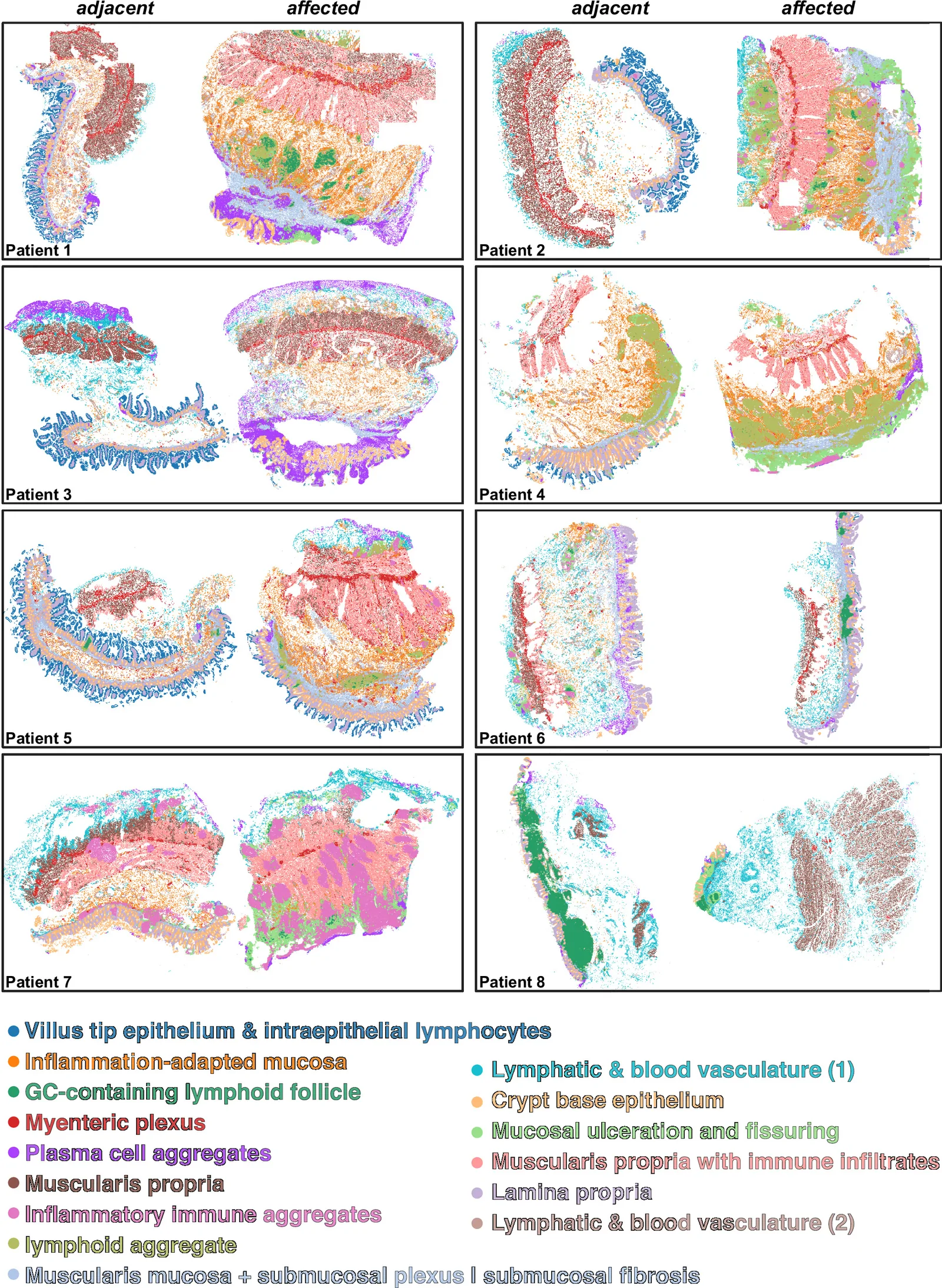
10X Xenium on-tissue cellular niches. On-tissue plots for each of the *n* = 16 CD patient tissues analyzed by Xenium 5 K spatial transcriptomics. Colors represent cellular niches derived from CellCharter analysis.

**Extended Data Fig. 3:**
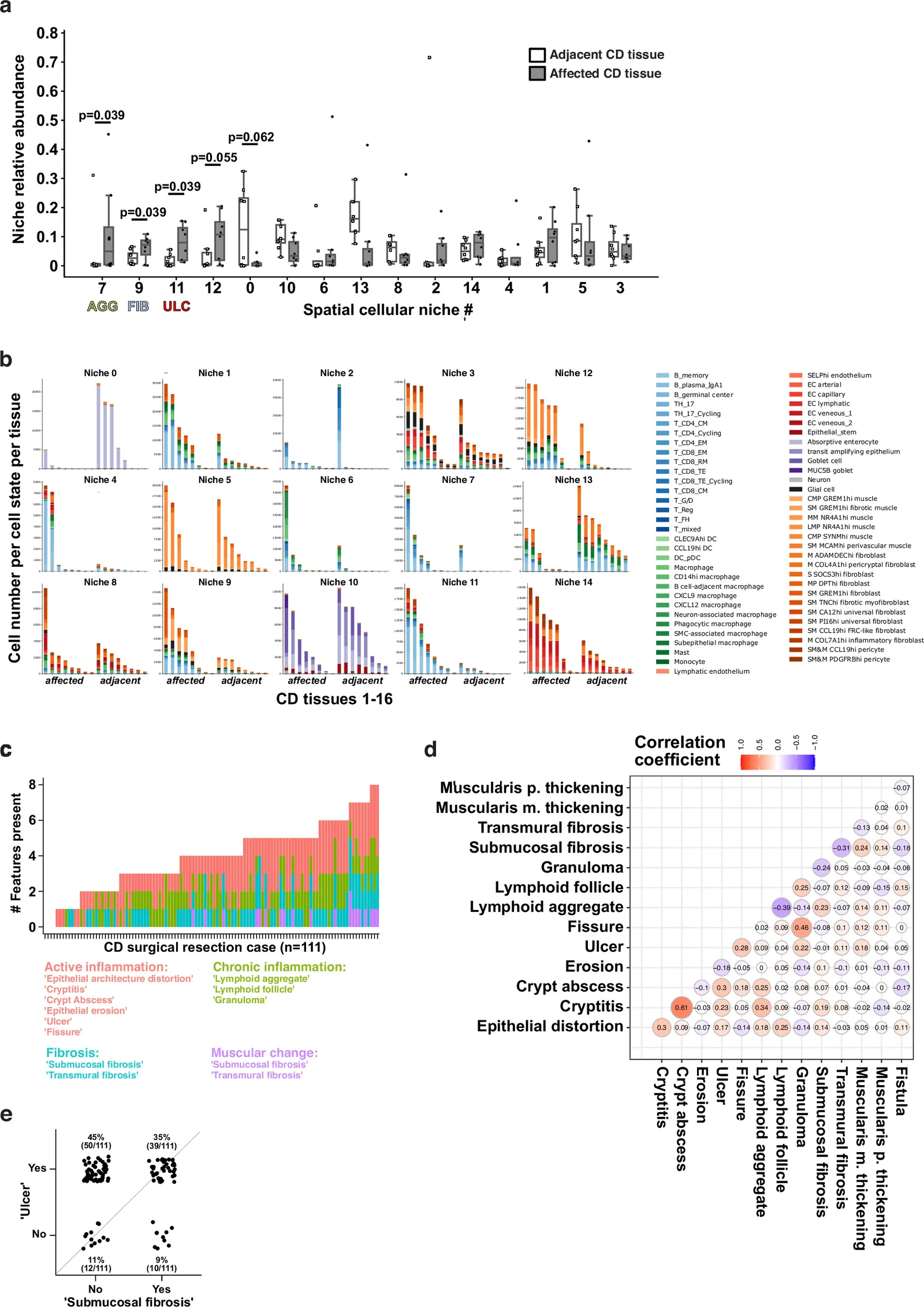
Crohn’s disease tissue cellular composition and histopathologic annotation. **a**, Boxplots of the relative abundance of niches in paired *n* = 8 adjacent vs n = 8 affected CD samples. Statistical significance of enrichment was evaluated with paired two-tailed Wilcoxon signed-rank test. Boxplots span 25^th^ to the 75^th^ percentile with whiskers extending to 1.5x of IQRs; colored horizontal bars indicate the median. **b**, Cell numbers per tissue for every niche across all n = 16 CD tissues profiled by 10x Xenium 5 K spatial transcriptomics; stacked barplot colors from bottom are in the order of the legend. **c**, Stacked barplots summarizing the counts of instances of different histopathologic features listed scored as present across n = 111 surgical CD specimen. **d**, Correlation plot of histopathologic features scored as in c; correlation coefficients (Pearson) were derived by converting the presence (1) or absence (0) of features into numeric values. **e**, Quadrant plot showing individual cases where ulcer or submucosal fibrosis have been scored as present or absent in the same tissue.

We set out to establish a spatial single-cell reference map of cell states within their pathologic tissue niches in CD, by adopting single-cell spatial transcriptomics (10x Xenium) using human Xenium Prime 5,000-plex panels, enabling broad cell type identification. To capture features of severe disease in CD, we selected *n* = 16 ileal tissues from *n* = 8 patients with longstanding disease and refractory to medical therapy (Supplementary Table 1). A surgical specimen, in contrast to mucosal biopsies, offers the advantage of capturing the entire thickness of the bowel wall. We identified broad epithelial, endothelial, stromal, lymphoid, myeloid and neuro-glial cell states, with stromal cells representing the largest fraction among the 2,131,098 detected cells (Fig. 1a). We first annotated cell states based on CellTypist label projection from a gastrointestinal (GI) reference atlas^17^, followed by manual refinement of the annotation, leveraging positional information revealed by Xenium spatial transcriptomics (Fig. 1a, Extended Data Fig. 1a,b and Supplementary Table 2). Using CellCharter^26^, we detected a total of *n* = 15 niches, reflecting cellular neighborhoods based on proximity and integrated gene expression (Fig. 1b and Supplementary Table 3). By contextualizing cellular composition (Fig. 1b) of niches with tissue localization (Extended Data Fig. 2), we annotated each niche as a morpho-cellular feature (Fig. 1b), relating to established histopathologist annotations (Fig. 1c). Niches included known homeostatic crypt base (niche 10) and villus epithelium (niche 0); muscularis propria (niche 5) and muscularis mucosa (niche 9); lymphatic and blood vasculature (niches 8 and 14); germinal center (GC)-containing lymphoid follicles (niche 2); lamina propria (niche 13); and glial cells and neurons of the enteric nervous system in the myenteric plexus (niche 3). In addition, we found maladapted pathological variants of these niches. In the affected tissue of some patients with CD (patients 1, 2, 4 and 7; Extended Data Fig. 2), crypt and villus epithelium were replaced by ulceration (niche 11) and lamina propria by lymphoid (niche 6) or plasma cell aggregates (niche 4). Patients 1–4 (Extended Data Fig. 2) demonstrated patches of submucosal fibrosis (niche 9), replacing or expanding the muscularis mucosa. Comparison of niche composition in the affected ileum to the adjacent tissue of the same patient identified pathologic niches that were significantly expanded (*P* < 0.05) (Extended Data Fig. 3a): lymphoid aggregates (‘AGG’) (niche 7), submucosal fibrosis (‘FIB’) (niche 9) and mucosal ulcers/fissures (‘ULC’) (niche 11).

In general, we observed heterogeneity in both the prevalence and abundance of these niches, as well as their cell state composition (Extended Data Fig. 3b), which was corroborated by pathologist scoring of CD surgical resection cases: the presence (y/n) of established histopathologic features of active inflammation, chronic inflammation, fibrosis and muscular changes varied substantially among the *n* = 111 cases analyzed (Extended Data Fig. 3c), with co-occurrence of scored histological features being weak to moderate (0.01–0.61 correlation coefficient; Extended Data Fig. 3d). While ulceration was found in the majority (80.2%) of tissues, submucosal fibrosis (44.1%) and muscularis mucosa thickening (11.7%) were observed less frequently, with co-occurrence in only 35% of cases (Extended Data Fig. 3e). This highlights that on a tissue micropathological level, the processes of ‘inflammation’ and ‘fibrosis’ (each of which can lead to the thickening of the bowel wall and indicate surgery) are spatially and cellularly distinct entities at an advanced disease stage.

Taken together, cellular niche profiling revealed a high heterogeneity of pathological tissue features in CD. ULC and FIB tissue niches are key features of inflammatory and fibrotic changes in affected CD tissue, which do not always co-occur. They form spatially separated structures, each with distinct stromal cell states dominating in the niche.

### Inflammatory fibroblasts reside in immune-rich microenvironments

**Fig. 2:**
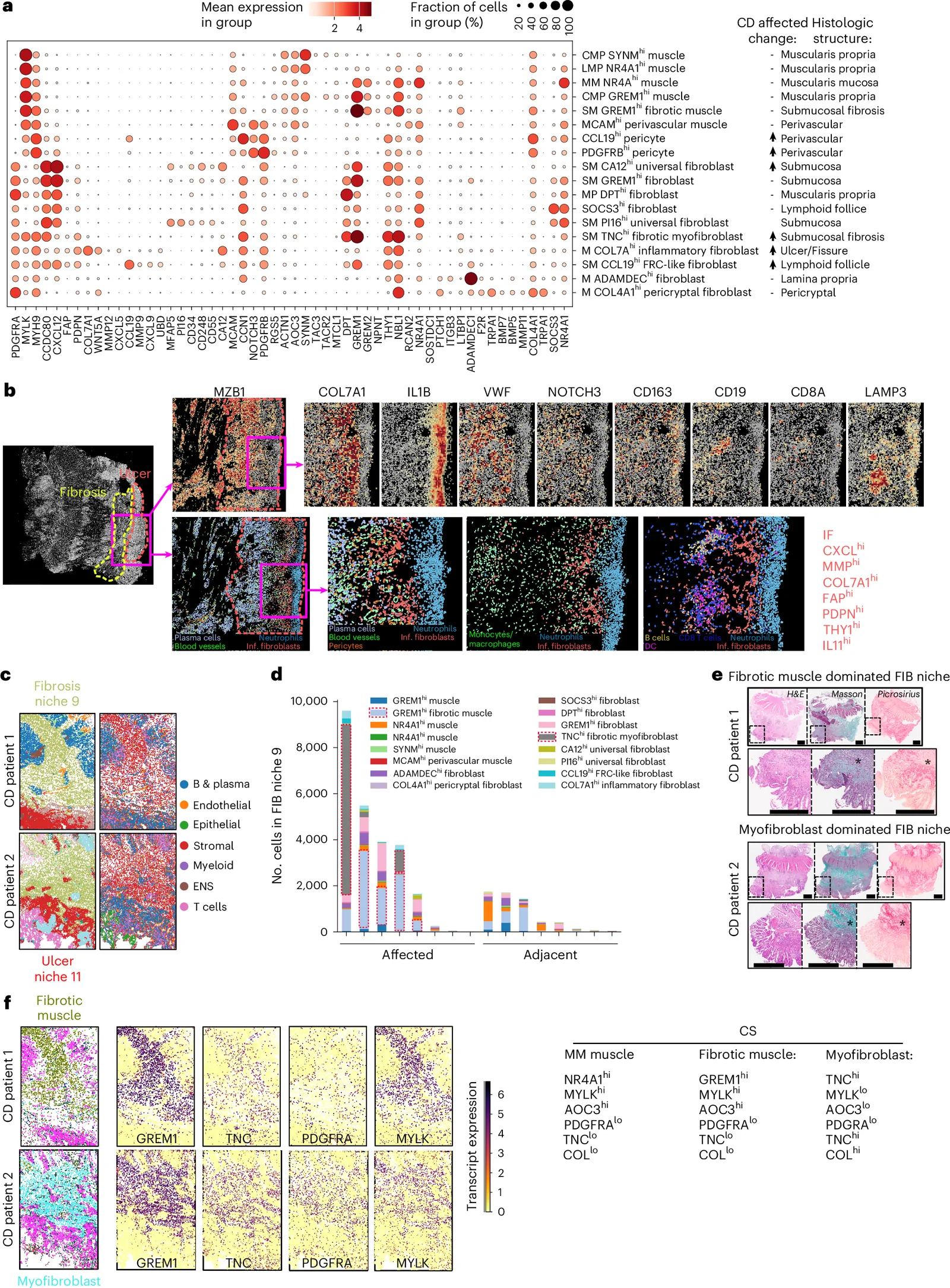
Inflammatory fibroblasts reside in immune-rich microenvironments with abundant and diverse cytokine signaling. **a**, Refined annotation of all stromal cell states detected by Xenium spatial transcriptomics across all affected and adjacent CD tissues. Expression of top markers for each stromal cell state are shown on dotplot. Clear association of the relative abundance of a stromal cell state in affected CD tissue is indicated (‘CD-affected change’), as is the location of the cell state within known histopathologic structures (‘histologic structure’). **b**, Serial section of the affected tissue of CD patient 2 that was profiled by 10x Xenium MultiTissue & Cancer panel with an add-on panel of 100 custom probes. Tissue-level overview (CD patient 2, tissue 3, affected) of areas of mucosal ulceration (ulcer, red) and submucosal fibrotic areas (fibrosis, ochre), indicating the margins of both (dotted line, approximation) (left). Top row shows the density of marker transcript expression in zoomed in areas of the same tissue (right). Bottom row shows cellular states in the environment of ulcer-associated inflammatory (inf.) fibroblasts. **c**, Cell lineage composition (right) of CellCharter niches 9 and 11 (left) identifying tissue areas of submucosal fibrosis FIB and mucosal ulceration ULC, respectively. **d**, Cell numbers per tissue within FIB niche 9 across all tissues (*n* = 16 CD) profiled by 10x Xenium 5k spatial transcriptomics; stacked barplot colors from bottom are in the order of the legend. Dotted red rectangles highlight whether myofibroblasts or fibrotic muscle (or both) dominate the niche in the individual tissue. **e**, Digital scans of either hematoxylin and eosin (H&E) (left), Masson’s trichrome (middle) or Picrosirius Red (right) chromogenic stains of serial sections from affected tissues of CD patients 1 and 2. * indicates areas of submucosal fibrosis with varying intensity of collagen staining across tissues. Scale bars, 1 mm. **f**, Different CS cell states (GREM1^hi^ fibrotic muscle and TNC^hi^ myofibroblasts) in fibrotic lesions as detected via 10x Xenium 5k spatial transcriptomics (left). Marker transcript expression within the same areas (tissue image zooms are the same as in **c**) (middle). Table summarizing identified markers in CS subtypes (right).

**Extended Data Fig. 4:**
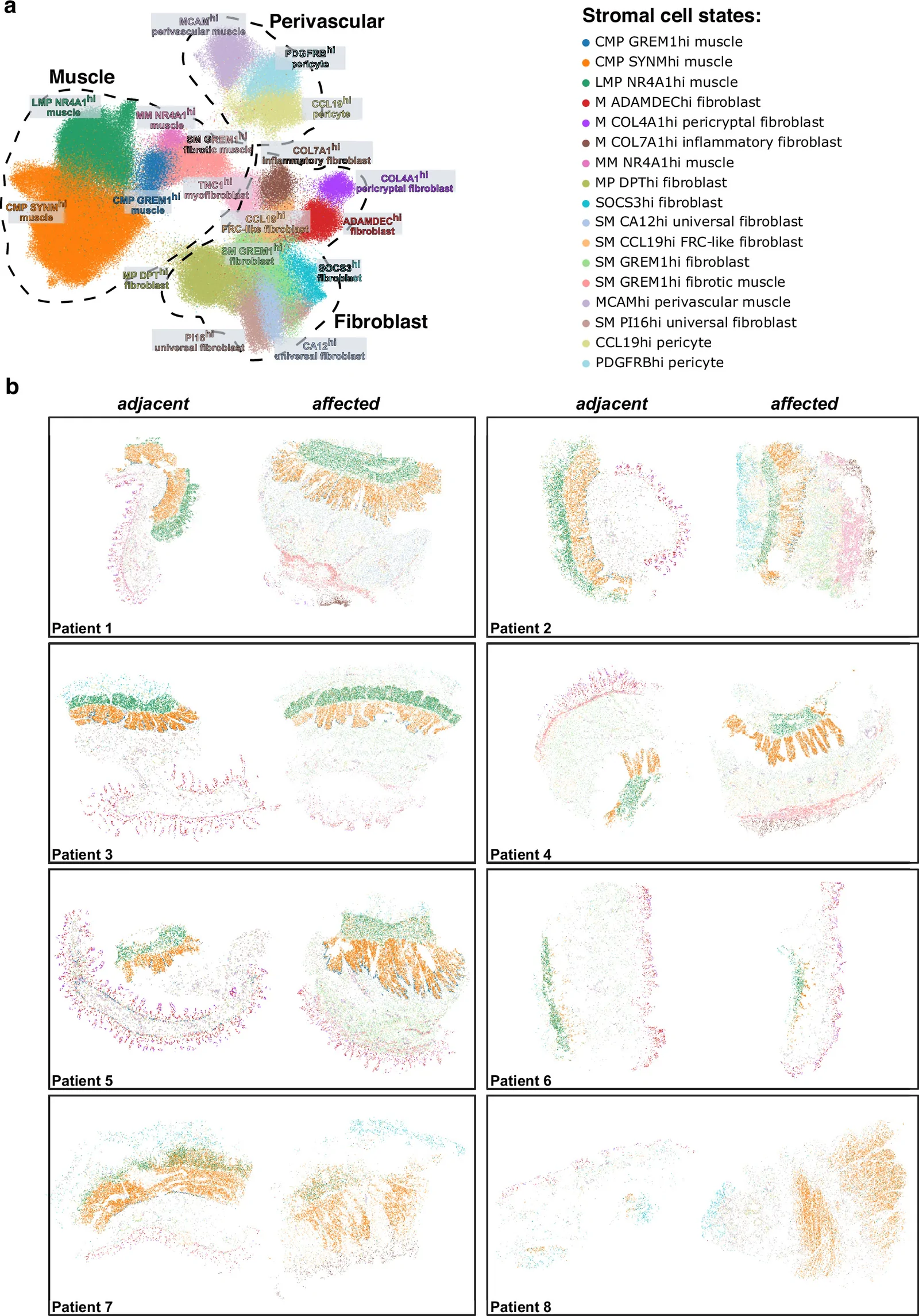
Stromal cell states and on-tissue locations. **a**, UMAP representation of all stromal cell states annotated in the Xenium 5 K spatial transcriptomics dataset consisting of n = 16 CD tissues; clear clustering for fibroblast- vs muscle- vs perivascular-like stromal states are indicated by dotted lines. **b**, On-tissue plots of the same color-coded stromal cells states as in a) across the n = 16 CD tissue analyzed by Xenium 5 K spatial transcriptomics.

COL7A1^hi^ inflammatory fibroblasts (‘IFs’) were the primary stromal cell state in ULC. By contrast, CS in the form of GREM1^hi^ muscle and TNC^hi^ myofibroblasts enriched in submucosal fibrotic areas, in addition to NR4A1^hi^ muscle found in healthy muscularis mucosa (Fig. 1b and Extended Data Fig. 3b). We further refined the annotation of the stromal cell lineage compartment (Fig. 2a) according to their functional property of fibroblast-like vs. muscle (Extended Data Fig. 4a) and their spatial location within the intestinal wall (Extended Data Fig. 4b). Where adjacent ulcers and submucosal fibrosis co-occurred, mucosal ulcers were composed toward the luminal side of a dense IL1B^+^ neutrophilic barrier, with a zone of COL7A1^hi^ IF stroma underneath (Fig. 2b). The local environment of IFs was further characterized by high vascularization (VWF^+^) with associated NOTCH3^+^ pericytes, the presence of CD163^+^ macrophages, and clusters of B cells, plasma cells, CD8^+^ T cells and DCs (Fig. 2b). By contrast, CS of the adjacent FIB niche formed part of an immune-depleted, stromal-rich microenvironment in submucosal fibrotic areas (Fig. 2c). Of note, we observed heterogeneity for the type of CS. Depending on the patient tissue, the FIB niche was either dominated by GREM1^hi^ fibrotic muscle or TNC^hi^ myofibroblasts (Fig. 2d), which correlated with collagen deposition: areas where fibrotic muscle was the dominating CS type showed lower staining intensity for Masson’s trichrome and Picrosirius Red (Fig. 2e) compared to areas where myofibroblasts were the predominant CS type (Fig. 2e). In line with this, GREM1^hi^ fibrotic muscle was characterized by high expression of GREM1 and MYLK, whereas TNC^hi^ myofibroblasts displayed high transcript levels for TNC and PDGFRA, but low expression of MYLK (Fig. 2f). This highlights that both stromal cell states are associated with fibrosis, but their relative abundance in the fibrotic niche may depend on the stage of the fibrotic remodeling process for the extent of contractility required.

**Fig. 3:**
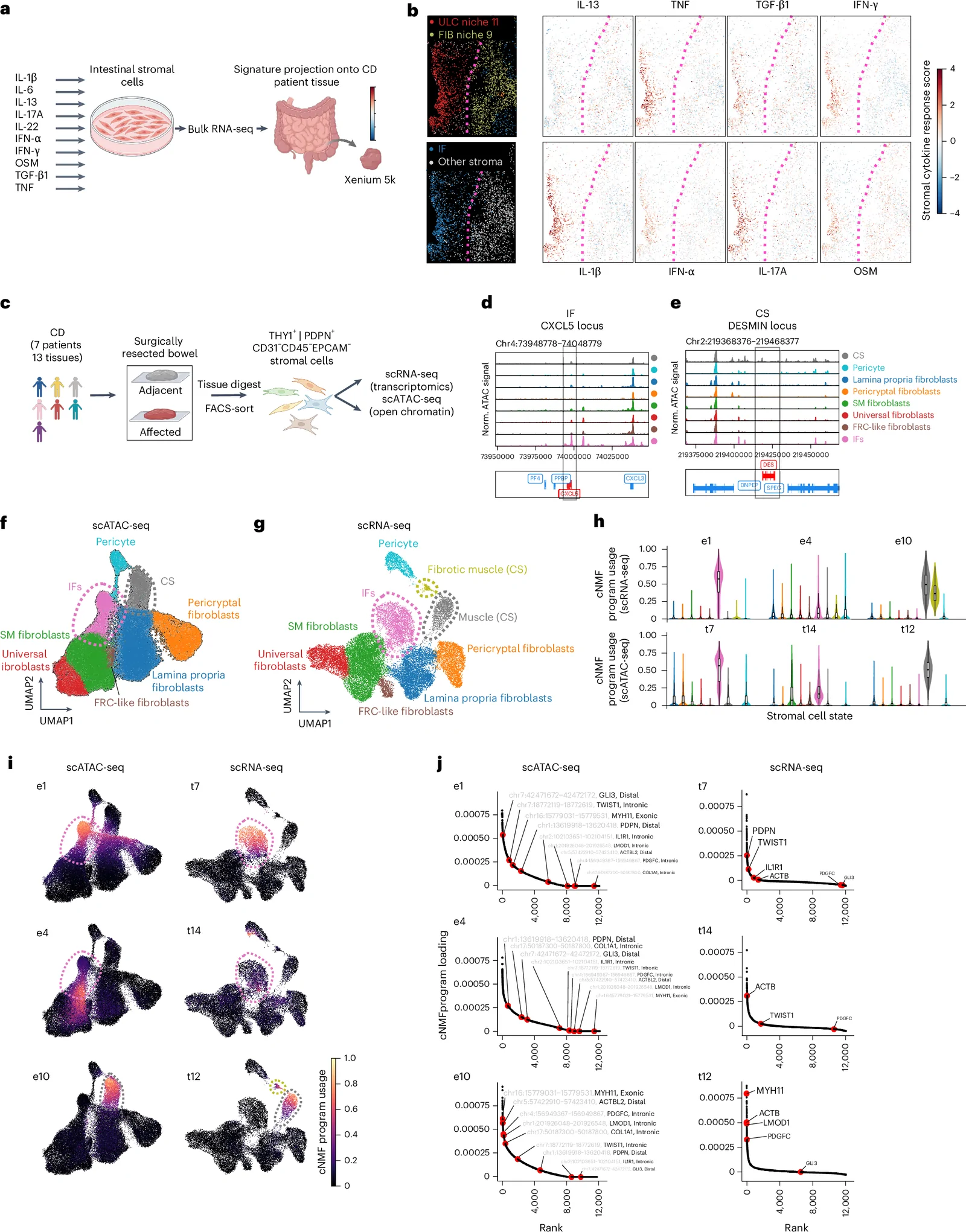
Inflammatory fibroblasts and contractile stroma display characteristic open chromatin configuration. **a**, Schematic overview of the experimental setup to derive bulk RNA-seq stromal cytokine response profiles in the human intestinal stromal cell line Ccd18-Co (*n* = 4 replicates per condition), and projecting signatures onto the 5k Xenium spatial transcriptomics dataset; with exception of IL-1β (0.01 ng ml^−1^), all cytokines were used at 10 ng ml^−1^ final concentration and cells were treated with cytokines for 24 h to generate signatures. **b**, Visualization of IFs residing in the mucosal ulceration niche, adjacent to the submucosal fibrosis niche (dotted magenta line indicates demarcation between ulcer and fibrotic niches, approximation) (left). Stromal cytokine response signatures generated as in **a**, scored onto 10x 5k Xenium spatial transcriptomics data and scores directly visualized on tissue 3 of CD patient 2 (right). **c**, Schematic of the profiling strategy for suspension scRNA-seq and scATAC-seq. Additional CD tissue samples (*n* = 13; patients 9–15, tissues 17–29) were profiled after enzymatic release of cells and pre-enrichment for the stromal compartment (as indicated; sorted on PDPN^+^ and/or THY1^+^) by FACS-sort. **d**,**e** Track plot representation of the coverage of scATAC-seq-derived reads in transcriptional start sites of selected genomic regions, split by annotated stromal cell state; DES, desmin. UMAP representation of clustered stromal cell states by open chromatin profiles; distinct inflammatory fibroblast and contractile muscle stroma cell states are highlighted (black circles). **f**, UMAP of stromal cell states clustered by chromatin accessibility based on scATAC-seq. **g**, UMAP of stromal cell states clustered by transcript expression based on scRNA-seq. **h**,**i**, Violin (**h**) and UMAP (**i**) representations of open chromatin cNMF program usage by IF (e1 and e4) and CS (e10) stromal cell states; these are aligned with the corresponding transcriptional cNMF programs. Boxes in violins span 25th to the 75th percentile with whiskers extending to 1.5× of interquartile range (IQR); horizontal bars indicate the median. **j**, cNMF program loadings (*y* axes) and ranks within programs (*x* axes) of features derived from either scATAC-seq or scRNA-seq: open chromatin peaks in genomic loci (left) and corresponding transcript expression (right). Panels **a** and **c** created in BioRender; Friedrich, M. https://biorender.com/o9ytcb2 (2026), licensed under CC BY 4.0 (https://creativecommons.org/licenses/by/4.0/).

**Extended Data Fig. 5:**
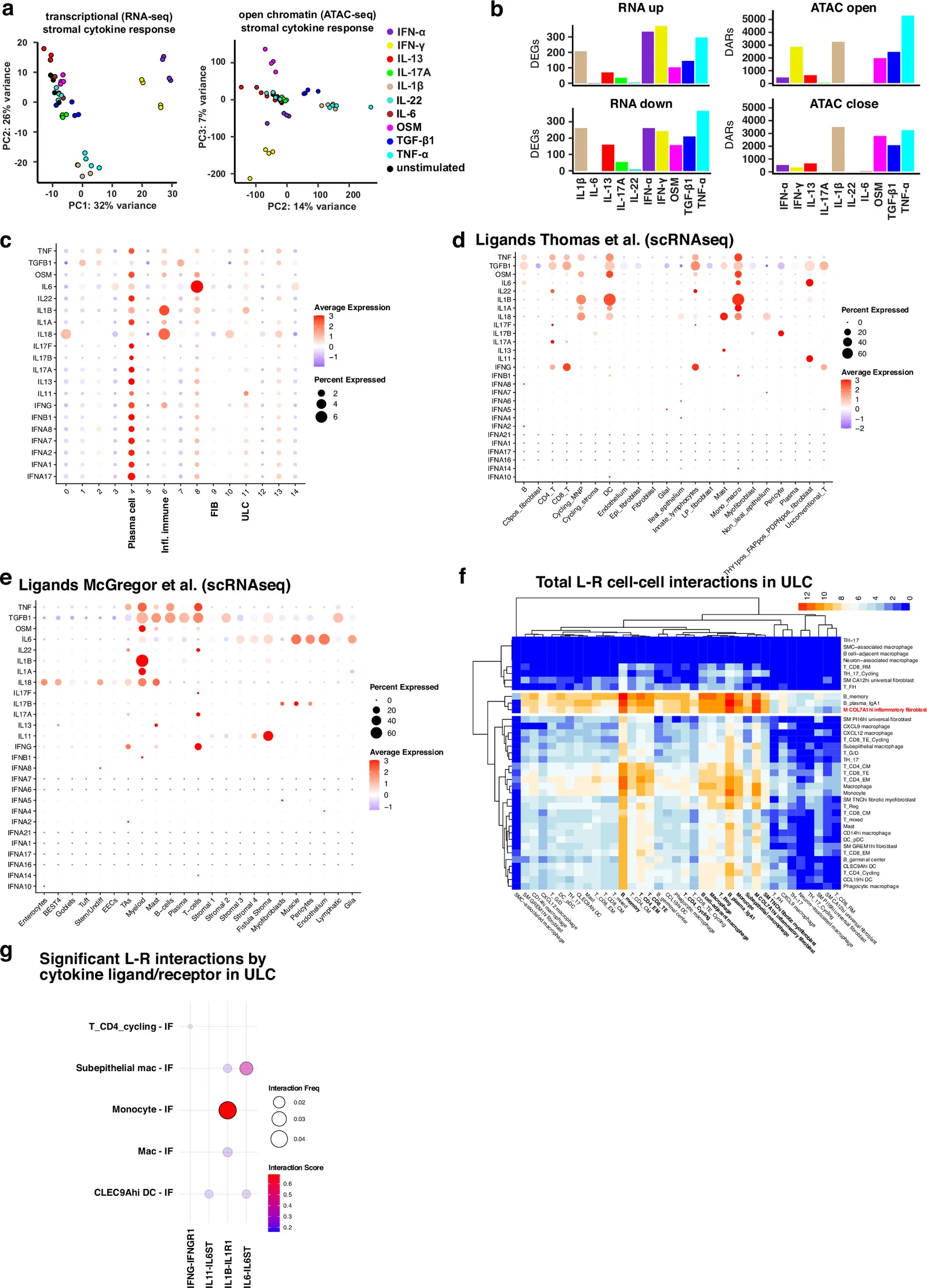
Stromal cytokine response profiles and ligand-receptor analyses. **a**, Principal component analysis (PCA) of the transcriptional and open chromatin profiles of Ccd18-Co human intestinal stromal cells 24 h after stimulation with various cytokines, derived from bulk RNA-seq or bulk ATAC-seq, respectively (n = 4 replicates per treatment group). All cytokines were used at 10 ng/ml, with the exception of IL-1b which was used at 0.01 ng/ml. **b**, Number of statistically significant (FDR < 0.05, fold change > 2) differentially upregulated or downregulated transcripts (pairwise two-sided Wald test) and open or closed chromatin regions (pairwise two-sided Wilcoxon) derived from the same experiment as in a). **c-e**, Dotplots highlighting proinflammatory cytokine expression across **(c)** Xenium 5 K niches (this study, n = 16 CD tissues), and scRNA-seq cell populations of CD samples from **(d)** Thomas et al. {Thomas, 2024 #229}, and **(e)** McGregor et al. {McGregor, 2026 #397}. **f**. Heat maps depicting total L-R based interactions between cell types in the ULC niche (n = 16 CD tissues). **g**. Dotplot highlighting significant L-R interactions from immune cells to IFs.

Given that IFs preferentially located to immune cell dense environments, it is likely that they are exposed to multiple soluble cytokines released from surrounding cells. To test this, we generated a bulk stromal cytokine response dataset (Fig. 3a) by treating an intestinal stromal cell line (Ccd-18Co) with cytokines previously associated with CD pathogenesis^1^: TNF, oncostatin M (OSM), IL-1β (IL-1), IL-13, IL-17A, IL-22, IFNα, IFNγ and TGF-β1. We extracted cytokine-specific transcriptional and epigenetic changes by bulk RNA sequencing (RNA-seq) and ATAC-seq after 24 h of stimulation. Generally, cytokines like IFNα and IFNγ, or TNF and IL-1β, elicited similarly strong overall effects on transcripts and open chromatin, whereas effects for cytokines like IL-13, IL-17A, TGF-β1 or OSM were less pronounced, but still distinct from unstimulated; IL-22 and IL-6 barely elicited any significant responses in stromal cells (Extended Data Fig. 5a, b). Differential gene expression (Supplementary Tables 4–13) and differential open chromatin (Supplementary Tables 14–23) between stimulated and unstimulated conditions defined cytokine-specific upregulated and downregulated signatures in stromal cells. Projecting these stromal cytokine response signatures onto 5,000-plex Xenium spatial data revealed that the highest cytokine signaling activity was concentrated in areas of IF in ulcers, compared to submucosal fibrotic regions where CS stroma is located (Fig. 3b). This was further highlighted by analysis of cytokine ligand expression across the 15 niches we had detected: ULC was among those niches (together with niches 4, 6, 8 and 13) with generally high cytokine ligand expression, including high *IL1*, *TNF*, *IL6* (*OSM* and *IL11*), *IL17*, IFN (type I and II) and TGF family cytokine expression (Extended Data Fig. 5c). Of note, the plasma cell niche demonstrated the highest expression of all cytokines analyzed, whereas inflammatory immune niche 6 showed a clear enrichment for *IL1B* and *IL18* (Extended Data Fig. 5c). Both niches are frequently found surrounding IFs in the ulcer niche (Extended Data Fig. 2), therefore IFs may be exposed to high cytokine signaling in the tissue surrounding the ulcer which might have led to their activation before ulcer formation. Analysis of additional single-cell CD-specific datasets confirmed the cellular sources of cytokine signaling (Extended Data Fig. 5d, e): *TNF* was most highly expressed by many immune cells, as well as *TGFB1* across immune and stromal cells; *IL1A*/*IL1B* and OSM showed highest expression in the myeloid compartment, whereas *IL17A* and *IFNG* were restricted to the T cell and innate lymphocyte compartments. As expected, fibroblasts (particularly IFs) showed highest expression of *IL11* and *IL6*, with the endothelial compartment overall constituting the highest expression of *IL6*. In corroboration, cell–cell interaction analysis of our spatial dataset inferred frequent interactions between IF and monocytes/macrophages, B/plasma cells, T cells and IF themselves specifically in the ULC niche (Extended Data Fig. 5f). Among these, *IL1B*–*IL1R1* signaling between monocytes and IF, as well as *IL6*–*IL6ST* signaling between macrophages and IF, were the most significant interactions (Extended Data Fig. 5g).

Taken together, our data demonstrate that IFs, in contrast to CS, reside in immune cell-rich niches of ULC with high localized and diverse cytokine signaling activity.

### IFs and CS have characteristic open chromatin profiles

Our findings suggested that specific microenvironments represented by maladapted ULC and FIB niches shape the transcriptional stromal cell states of IF and CS. Changes in transcriptional states are often accompanied by the modulation of chromatin accessibility through epigenetic regulation. To test this, we conducted scATAC-seq on additional CD tissues, matching to parallel scRNA-seq for referencing it to transcriptional stromal cell states. We profiled *n* = 7 affected and *n* = 6 adjacent resected tissues from patients with CD (Table S1) undergoing surgery. To obtain a high-resolution map of stromal cell states, we FACS-sorted tissue digests to enrich for THY1^+^|PDPN^+^EPCAM^−^CD45^−^CD31^−^ populations before profiling by suspension scRNA-seq and scATAC-seq (10x) (Fig. 3c).

**Extended Data Fig. 6:**
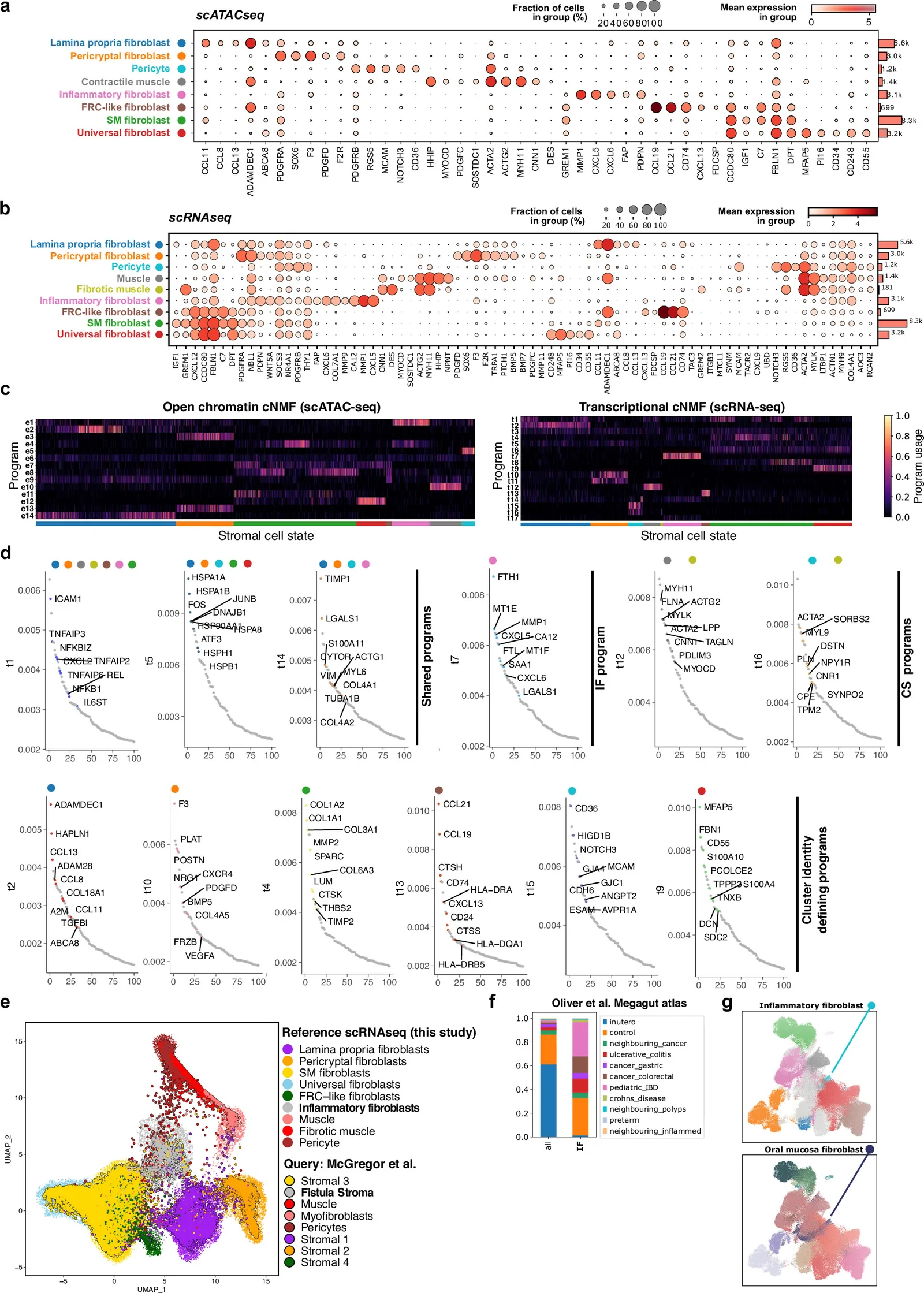
10X single-cell RNAseq and single-nuclei ATACseq annotations and cNMF programs. **a, b**, Dotplots of top marker open chromatin peaks coalesced into ArchR gene scores and (**b**) transcripts for stromal cell states obtained by 10x chromium scATAC-seq (**a**) and tanscripts for stromal cell states obtained by 10X chromium scRNA-seq, respectively (**b**; CD patients 9-15, tissues 17-29). Barplots indicate total number of nuclei or cells per cluster. **c**, Heatmap representation of transcriptional cNMF program usage per stromal cell state (color-coded horizontal bar below x-axis). **d**, Top-100 (ranks on x-axes) transcriptional cNMF factor loadings (y-axes) for programs shared and unique to stromal cell states. **e**, UMAP representation with original annotations (scRNA-seq data from McGregor et al. {McGregor, 2026 #397}) projected onto the annotated stromal scRNA-seq reference dataset of this study. **f**,**g**, Barplot (**f**) and UMAP (**g**) representations with IF label predictions in the pan-GI atlas {Oliver, 2024 #230}; Barplot (**f**) shows relative proportions of enriched IF label predicted cell states in a range of GI diseases and organs; UMAP (**g**) shows IF label prediction in the same dataset, which aligns with the original label of ‘oral mucosa fibroblasts’ in the Oliver et al. dataset.

scATAC-seq and scRNA-seq of these tissues resulted in eight clusters by open chromatin profiles (56,689 nuclei) (Extended Data Fig. 6a and Table S24), corresponding by label transfer to nine clusters by transcriptional profiles (26,727 cells) (Extended Data Fig. 6b and Table S25). By scATAC-seq, we detected characteristic open chromatin peaks in key marker loci of stromal cell states: chemoattraction (for example CXCL5) for IF (Fig. 3d) and contractility (DESMIN) for CS (Fig. 3e). Notably, the distinction between IF and CS stromal states prevailed when clustered on open chromatin profiles (Fig. 3f, g). Next, we inferred single-cell open chromatin and transcriptional programs using consensus non-negative matrix factorization (cNMF)^27^. cNMF programs on both scRNA-seq (t1–t17; Table S26) and scATAC-seq (e1–e14; Table S27) datasets showed that most transcriptional and open chromatin clusters were associated with a stromal cell state-defining program, while some programs were shared between states (Extended Data Fig. 6c). For example, IFs were characterized by program t7 reflecting matrix remodeling (for example, MMPs) and chemoattraction (for example, CXC-type chemokines), but shared matrix (for example, COL4A1/A2) and contractility (for example, ACTG1) program t14 with other stromal cell states (Extended Data Fig. 6d). Notably, the composition of this contractility program used by IF differed from the contractile myosin-actin programs t12 and t16 (for example, ACTA2, MYLK) unique to CS states (fibrotic muscle and muscle) and pericytes (Extended Data Fig. 6d). Similar to these, open chromatin programs e1 and e4 were highly used by IF, and program e10 used by CS (Fig. 3h, i).

Of note, open chromatin loci in GLI3, TWIST1, PDPN and IL-1R1 were consistently among the top-ranked contributing features to IF programs e1 and e4, which was mirrored by the contribution of PDPN, TWIST1 and IL-1R1 transcripts to transcriptional programs t7 and t14 (Fig. 3j). Compared to PDPN, GLI3, TWIST1 and IL-1R1, the CS-characteristic program e10 showed increased contribution of open chromatin loci in contractile (ACTBL2) and matrix/remodeling (LMOD1) genes, as well as PDGFC known to promote survival, proliferation and migration of muscle cells and fibroblasts (Fig. 3j).

High PDPN and IL-1 signaling are established hallmarks of IF^11^, and TWIST1 has been recently identified as key TFs to promote IF-like states in Crohn’s fistula^21^. Indeed, integration of our single-cell dataset with the Crohn’s fistula atlas^21^ showed that the described fistula-associated stromal population maps to the IF stromal cell state we detected in mucosal ulcers and deeper fissures (Extended Data Fig. 6e). Moreover, stromal cell states resembling IF were also found in ulcerative colitis and colorectal cancer of a pan-gastrointestinal atlas^17^ (Extended Data Fig. 6f), including healthy oral mucosa fibroblasts of the gingiva (Extended Data Fig. 6g) known to form pre-inflammatory niches^28^.

**Extended Data Fig. 7:**
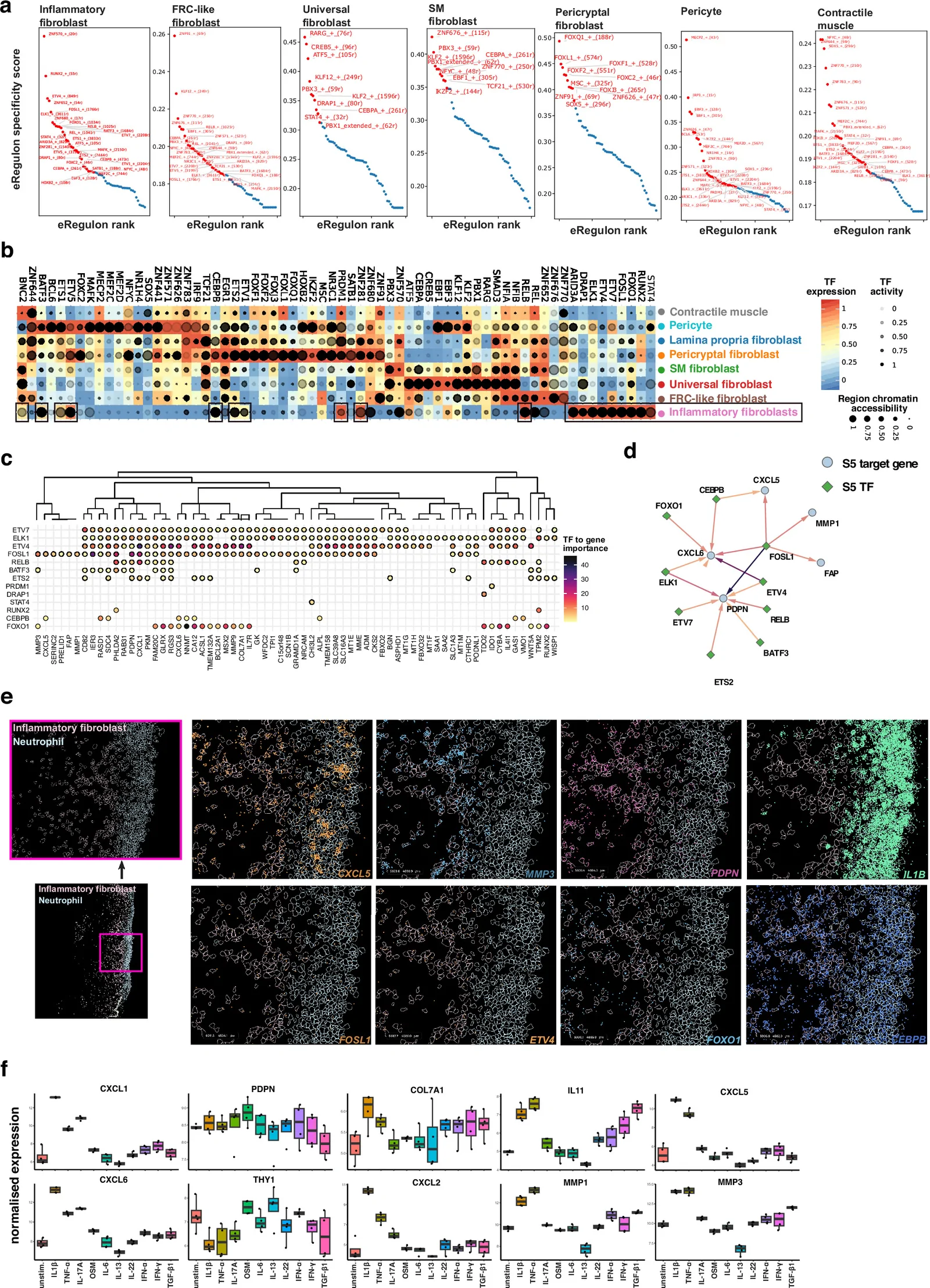
Stromal cell state regulon network analyses. **a**, Top eRegulons obtained by SCENIC+ gene regulatory network inference for each stromal cell state. **b**, Heatmap of SCENIC+ derived transcription factors (TF) in the top-scored regulons per stromal cell state; color-coding represents TF expression level (transcript), gray-shade coding represents TF activity for its target gene sets; dot size represents regulon region accessibility determined by ATAC-seq. **c**, Heatmap of transcription factor (TF) to target gene importance for top IF TFs (y-axis) and marker genes (x-axis). **d**, Schematic of a putative gene regulatory network of key regulons for IF. **e**, 10x Xenium 5 K spatial transcriptomics-based localization of transcripts for IF and neutrophil markers (upper row) and transcription factors (lower row) within detected IFs (red) or neutrophils (blue) in ulceration tissue of CD patient 2. **f**, Boxplot representation of bulk RNA-seq-derived, variance stabilizing transformation-normalized transcript expression in Ccd18-Co cell line upon 24 h stimulation with individual cytokines as shown (n = 4 replicates per cytokine treatment group). Boxplots span 25^th^ to the 75^th^ percentile with whiskers extending to 1.5x of IQRs; horizontal bars indicate the median.

Given the distinct open chromatin configuration of IF and CS, we sought to identify associated regulons (the TFs and genomic target loci they bind). To this end, we integrated scATAC-seq open chromatin profiles with scRNA-seq transcriptional profiles using modality integration through MultiMAP^29^ (Supplementary Fig. 1a,b), followed by matching RNA and ATAC cells within each stromal cell state using SEACell metacell^30^ (Supplementary Fig. 1c). To achieve within-cell state integration accuracy, we integrated cells per cell type while excluding metacells with fewer than 10% cross-platform matches (Supplementary Fig. 1d). This integration formed the input for SCENIC^+^^(ref. 31^) analysis inferring TF regulons based on three layers of evidence: expression level (transcript) of TFs; chromatin region accessibility in target gene (open chromatin); and the predicted activity of a TF on target genes (Supplementary Tables 28–30). Characteristic TF regulons were observed for most stromal cell states (Extended Data Fig. 7a). Most notably, the IF stromal cell state was characterized by a unique set TFs (Extended Data Fig. 7b). Among these, predictions of RUNX2, FOXO1, FOSL1, ETV7, ETV4, ELK1, DRAP1, ARID3A and RELB TFs were supported by both RNA (expression level and TF activity) and ATAC layers (region chromatin accessibility); however, open chromatin accessibility derived from the ATAC data highlighted additional potential TFs including BNC2, ETS1, CEBPB, ETS2 and ETV1 (Extended Data Fig. 7b). Of note, these IF-specific TFs did not show high activity in CS, emphasizing the distinct nature of IF and CS on both transcriptional and chromatin accessibility level. Visualizing the network of TF candidates for regulating specific IF marker target gene sets revealed overlapping and non-overlapping functions: CXCL5, FAP and MMP1 were predicted to be regulated by FOSL1 and CEBPB, whereas CXCL6 and PDPN are predicted to be overlappingly regulated by a range of TFs (Extended Data Fig. 7c,d). Localizing these TFs in the tissue revealed that FOSL1 and ETV4 were specifically expressed in IFs in the ulcer area (Extended Data Fig. 7e). Other TFs like FOXO1 and CEBPB were located in IF, but also in surrounding cells such as neutrophils (Extended Data Fig. 7e).

In summary, our data support a model where maladapted ULC and FIB tissue niches have shaped stromal cell states of IFs and CS, which each display distinct and characteristic open chromatin profiles and TF activity. IF-like states are also found in other diseases and organs of the GI tract which feature inflammatory or pre-inflammatory microenvironments, suggesting a common pathway that might generate this stromal cell state.

### Combinatorial cytokine stimulation generates a stable IF state

**Fig. 4:**
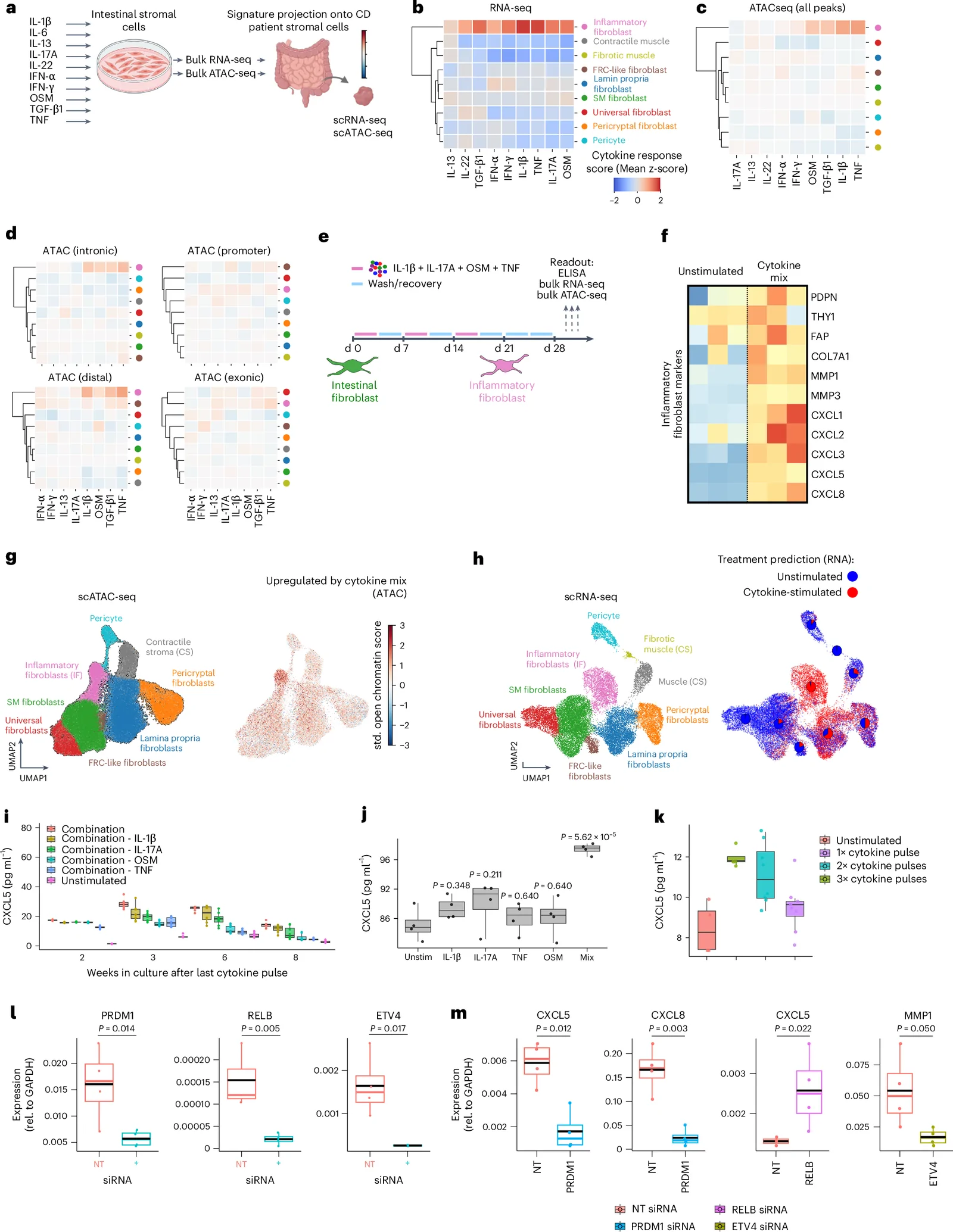
Combinatorial cytokine stimulation generates a stable IF state. **a**, Stromal cytokine response signatures generated by bulk RNA-seq and bulk ATAC-seq in Ccd18-Co as in Fig. 3a). **b**–**d**, These were scored on to 10x Chromium suspension scRNA-seq (**b**) or scATAC-seq data (**c**,**d**) of stromal cell states. **e**, Schematic overview of the experimental setup to generate IFs in vitro. The human intestinal stromal cell line Ccd18-Co was subjected to three pulses of a cytokine mix containing IL-1β (0.01 ng ml^−1^), OSM (10 ng ml^−1^), IL-17A (10 ng ml^−1^) and TNF (10 ng ml^−1^). After the last pulse, fibroblasts were rested for at least 2 weeks before the generation of bulk RNA-seq (*n* = 3 replicates per treatment group), bulk ATAC-seq (*n* = 6 replicates per treatment group) or ELISA. **f**, Heatmap of select IF markers (normalized transcript counts, RNA-seq, *n* = 3 replicates per treatment group) 2 weeks after the last pulse with the cytokine mix, experimental setup as in **e**). **g,** in vitro generated IF bulk ATAC-seq signature scored on CD patient tissue-derived suspension scATAC-seq. Only scores for upregulated open chromatin peaks are used for the scoring. **h**, UMAP of scRNA-seq stromal cell states (as in Fig. 3g), colored by *k*NN-based classification using bulk RNA-seq profiles of unstimulated and cytokine-mix-stimulated Ccd18-Co cells as reference. Each cell is assigned to the most transcriptionally similar condition: blue cells represent cells assigned to unstimulated cells, red to cytokine-stimulated cells, and cells with low-confidence prediction are colored in gray (NA, cosine similarity < 0.3). Pie charts indicate the proportion of cells assigned to each condition within each annotated stromal cell state. **i**, Results of in vitro IF polarization, measured by ELISA-based quantification of secreted hCXCL5 in culture supernatants 2, 3, 6 and 8 weeks after the last cytokine pulse. For some conditions, one of the four cytokines was omitted from the mix in the phase where Ccd18-Co are subjected to the cytokine pulses (*n* = 8 replicates per condition). Boxplots span 25th to the 75th percentile with whiskers extending to 1.5× of IQRs; horizontal bars indicate the median. **j**, Results of in vitro IF polarization, measured by ELISA-based quantification of secreted hCXCL5 in culture supernatants 2 weeks after the last cytokine pulse. Either one of the four cytokines or the combination of all four cytokines were used for polarization (*n* = 4 biological replicates per condition; false discovery rate (FDR) derived from pairwise two-sided *t*-tests for the comparison with the unstimulated condition). Boxplots span 25th to the 75th percentile with whiskers extending to 1.5× of IQRs; horizontal bars indicate the median. **k**, Results of in vitro IF polarization, measured by ELISA-based quantification of secreted hCXCL5 in culture supernatants 2 weeks after either one, two, or three pulses with the cytokine mix (*n* = 4 replicates for unstimulated and 1× cytokine pulse condition; *n* = 8 replicates fpr 2× and 3× cytokine pulse conditions). Boxplots span 25th to the 75th percentile with whiskers extending to 1.5× of IQRs; horizontal bars indicate the median. **l**,**m**, Results of in vitro IF polarization followed by siRNA knockdown of RELB, PRDM1 and ETV4 TFs. Transcript expression relative to GAPDH (2^Δct^) is shown; *n* = 4 biological replicates per condition; *P* values derived from pairwise two-sided *t*-tests for comparisons to the control condition using nontargeting (NT) siRNA. Boxplots span 25th to the 75th percentile with whiskers extending to 1.5× of IQRs; colored horizontal bars indicate the median, black horizontal bars indicate the mean. Panel **a** created in BioRender; Friedrich, M. https://biorender.com/o9ytcb2 (2026), licensed under CC BY 4.0 (https://creativecommons.org/licenses/by/4.0/).

Our spatial profiling had identified IFs in areas of high immune cell density and cytokine signaling, suggesting that these may represent the specific environmental cues that generate and maintain their state. We first projected our stromal cytokine response signatures onto our 10x Chromium suspension scRNA-seq and scATAC-seq data (Fig. 4a). This confirmed that the strongest cytokine response on transcriptional (Fig. 4b) and chromatin accessibility level (Fig. 4c) in IFs among all stromal states, predominantly characterized by changes in intronic and distal enhancer regions (Fig. 4d).

To test experimentally whether IF can be generated by combinatorial cytokine signaling, we subjected the intestinal stromal cell line Ccd18-Co to three repeated rounds of stimulation with a cytokine cocktail composed of TNF, OSM, IL-1β and IL-17A, generating RNA-seq-based signatures after a resting period (>2 weeks) after cytokine exposure (Fig. 4e). The choice of these four cytokines was based on their capacity to induce IF marker expression (Extended Data Fig. 7f): IL-1β most strongly induced CXC-type chemokines and COL7A1 expression; TNF most strongly upregulated MMPs 1/3 and IL-11; OSM most strongly upregulated PDPN and THY1; and IL-17 was chosen as it is well established to synergize with IL-1β or TNF for amplifying gene expression through CEBPB^32^, a TF that we had identified to be highly expressed in IFs (Extended Data Fig. 7b). To assess the extent by which in vitro treated stromal cells after the three cytokine pulses resemble the tissue IF state, we first assessed IF marker transcript expression: key marker transcripts of IF for activation (PDPN, THY1 and FAP), matrix remodeling (COL7A1, MMP1 and MMP3) and chemotaxis (CXCL1/2/3/5/8) were upregulated (Fig. 4f). The repeated exposure to combinatorial cytokines also resulted in the upregulation of an open chromatin profile closely resembling the IF stromal state observed in tissue from patients with CD (Fig. 4g). Scoring signatures of unstimulated Ccd18-Co on our scRNA-seq data revealed highest scores in non-IF stromal cell states (Fig. 4h), whereas fibroblasts after cytokine pulses scored highest in IF (Fig. 4h). This confirmed that the resting cell line was not already pre-polarized toward the IF state and that it reflects homeostatic states instead. Each of the four cytokines was required to achieve the maximum effect, and that the IF state is stable in vitro for more than 8 weeks after the last cytokine pulse (Fig. 4i). Similarly, TNF, OSM, IL-1β and IL-17A individually did not substantially induce an IF state, but the combination of all four cytokines was required (Fig. 4j), as was the chronic stimulation by three repeated exposures to the cytokine mix to achieve maximum effect (Fig. 4k).

Given we had identified a set of putative TFs active in IFs (Extended Data Fig. 7b), we sought to validate experimentally that these are pivotal to maintain the IF phenotype in vitro. siRNA-mediated knockdown of several of these TFs (Fig. 4l) revealed counterregulatory functions: knockdown of PRDM1 reduced CXC-type chemokine expression, whereas knockdown of RELB increased it (Fig. 4m). Knockdown of ETV4 reduced MMP1 transcript levels in IFs in vitro (Fig. 4m).

In summary, our data provides experimental evidence that the IF state can be generated in vitro by repeated exposure to combinatorial cytokine pulses, mimicking the immune-rich microenvironment in ulcerated patient tissue which is missing in adjacent immune-depleted fibrotic areas. Cytokine exposure results in a characteristic change in open chromatin configuration, leading to a stable IF state that does not revert spontaneously even after months in culture. Silencing TFs predicted to be particularly active in IF differentially regulates IF functional marker gene expression.

### Universal fibroblasts and myofibroblasts are likely precursors to IFs

**Fig. 5:**
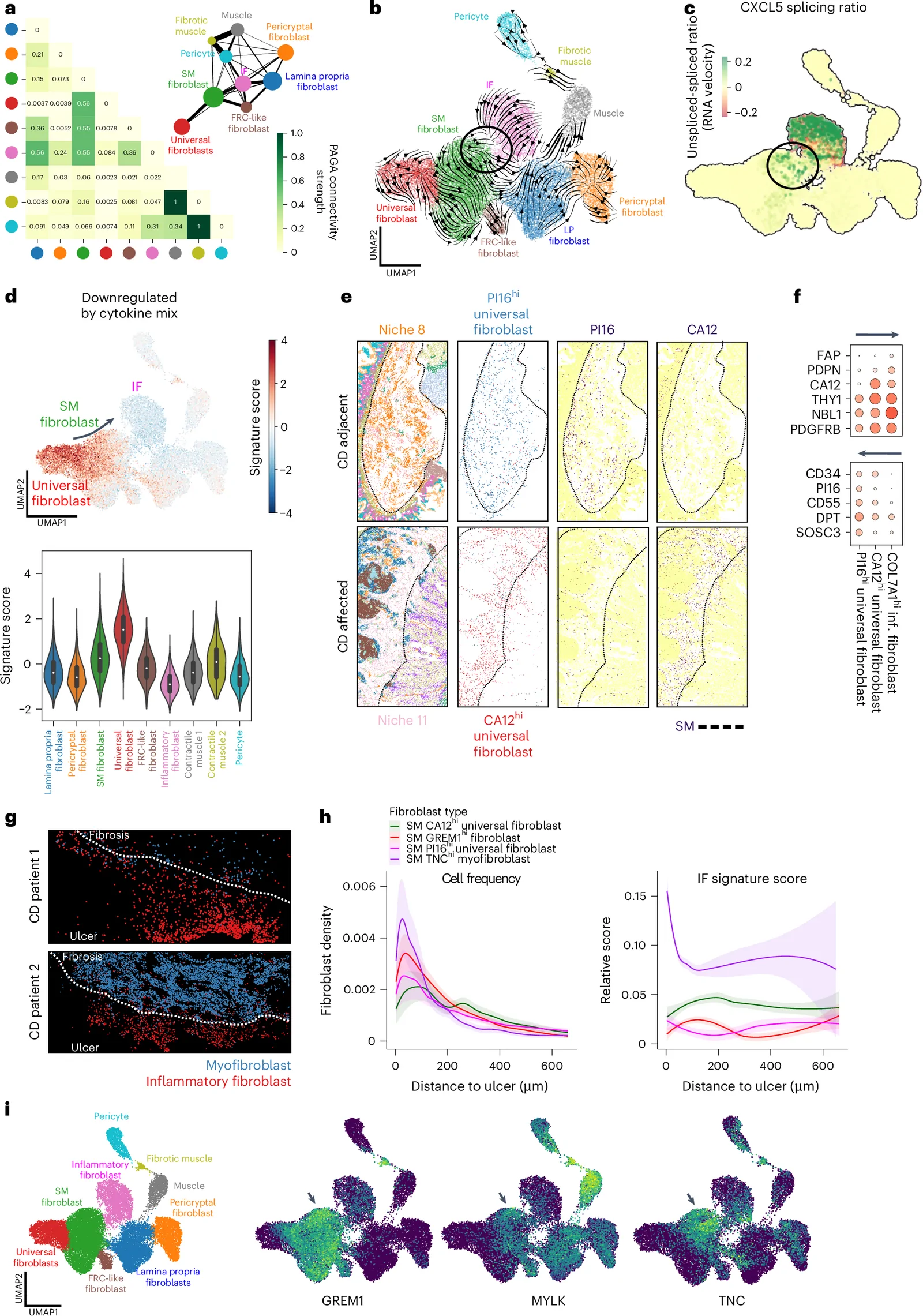
Submucosal universal fibroblasts and myofibroblasts are likely tissue precursors to IFs. **a**, PAGA connectivity of stromal cell states based on 10x Chromium suspension scRNA-seq profiling data (patients 6–12, tissues 11–23). **b**, RNA velocity analysis of stromal cell states based on 10x Chromium suspension scRNA-seq profiling data; black arrows indicate locally averaged RNA velocity field. **c**, Splicing ratios of the CXCL5 transcript shown on the UMAP representation of suspension scRNA-seq profiling data. **d**, In vitro generated IF bulk RNA-seq signature of downregulated genes (*n* = 3 replicates per treatment group; log_2_ fold change > 2; adjusted *P* value < 0.05; DESeq2) scored on CD patient tissue-derived suspension scRNA-seq data. Top shows scoring (color code) of the in vitro IF signature on stromal clusters represented as UMAP, bottom as violin plots of the score. Only scoring for transcripts downregulated after IF priming is shown. Boxes in violins span 25th to the 75th percentile with whiskers extending to 1.5× of IQRs; horizontal bars indicate the median. **e**, Changes of submucosal universal fibroblasts in affected CD tissue. Overview of submucosal CellCharter niches (1 and 15), universal fibroblast states (CA12^hi^ and PI16^hi^), and key markers (CA12 and PI16) in areas of the submucosa (dotted line, approximation). Data based on 10x 5k Xenium spatial transcriptomics profiling as in Fig. 1. **f**, Dotplot representation of the continuum from PI16^+^ over CA12^+^ universal fibroblasts toward IF, based on 10x 5k Xenium spatial transcriptomics. IF markers gradually upregulated along this trajectory (top) and universal fibroblast markers downregulated (bottom). **g**, IFs and myofibroblasts localize close to each other in adjacent ulceration and fibrotic niches; localization based on 10x 5k Xenium spatial transcriptomics data. **h**, Nearest-neighbor distance analysis from submucosal fibroblast populations (PI16hi and CA12^hi^ universal fibroblasts, GREM^hi^ fibroblast and TNC^hi^ myofibroblasts) to the ULC niche, highlighting the increased proportion of TNC^hi^ myofibroblast frequency (left) and IF-like signature expression (right) closer to the ULC niche. Error bars indicate s.e.m. **i**, Myofibroblast marker gene expression (normalized) within the submucosal fibroblast cluster, based on suspension scRNA-seq data.

Having identified the cytokine cues that stably generate IFs, we next explored their potential tissue-resident stromal precursor populations. Determining connectivity between the different stromal states detected by our suspension scRNA-seq using partition-based graph abstraction (PAGA) analysis^33^ revealed the strongest connections within CS states of pericytes and muscle (Fig. 5a). Similarly, strong connectivity was observed between universal, submucosa (SM), IF, lamina propria and fibroblastic reticular cell (FRC)-like fibroblast states (Fig. 5a). RNA velocity analysis predicted a general precursor state of universal fibroblasts to these (in line with previous reports^34^), with directionality flowing outward into the SM fibroblasts, and from there into subsets of IF and lamina propria fibroblast stroma (Fig. 5b). Moreover, despite an extensive 2,013-bp exon 4 at the polyA tail, the spliced-unspliced ratio of CXCL5 transcripts supported IF precursors within the SM fibroblast population (Fig. 5c) (Fisher’s exact test, odds ratio (OR) = 15.56, *P* < 0.001). Cytokine perturbation experiments carried out as shown in Fig. 4e further support this cellular origin, showing that the combinatorial cytokines inducing IF downregulate signatures of universal and SM fibroblasts (Fig. 5d). Furthermore, we noticed a gradual remodeling of the submucosal fibroblast compartment, comprising a switch from PI16^hi^ (niche 8) to CA12^hi^ (niche 1) universal fibroblast states, consistent with inflammatory cytokine remodeling suppressing the PI16^hi^ state (Fig. 5e, f).

IFs in mucosal ulcer regions are found directly adjacent to submucosal fibrotic regions where myofibroblasts reside (Fig. 5g), so we asked where TNC^hi^ myofibroblasts locate in the trajectory determined above. Spatial proximity analysis revealed that TNC^hi^ myofibroblasts both adopt a more IF-like signature and proportionally increase in frequency the closer they are to the ulcer niche (Fig. 5h). Plotting key activation (THY1 and TNC) and contractile (MYLK and GREM1) markers of the myofibroblast population identified a subpopulation within SM fibroblasts, located closely to IF on the Uniform Manifold Approximation and Projection (UMAP) (Fig. 5i) where we also had detected altered CXCL5 splicing ratios (Fig. 5c).

Overall, these findings support a model where IFs originate from submucosal universal fibroblasts through exposure to combinatorial cytokine signaling. IFs and myofibroblast display close ontogenic relationships.

### Epigenetic modulation destabilizes the IF state

**Fig. 6:**
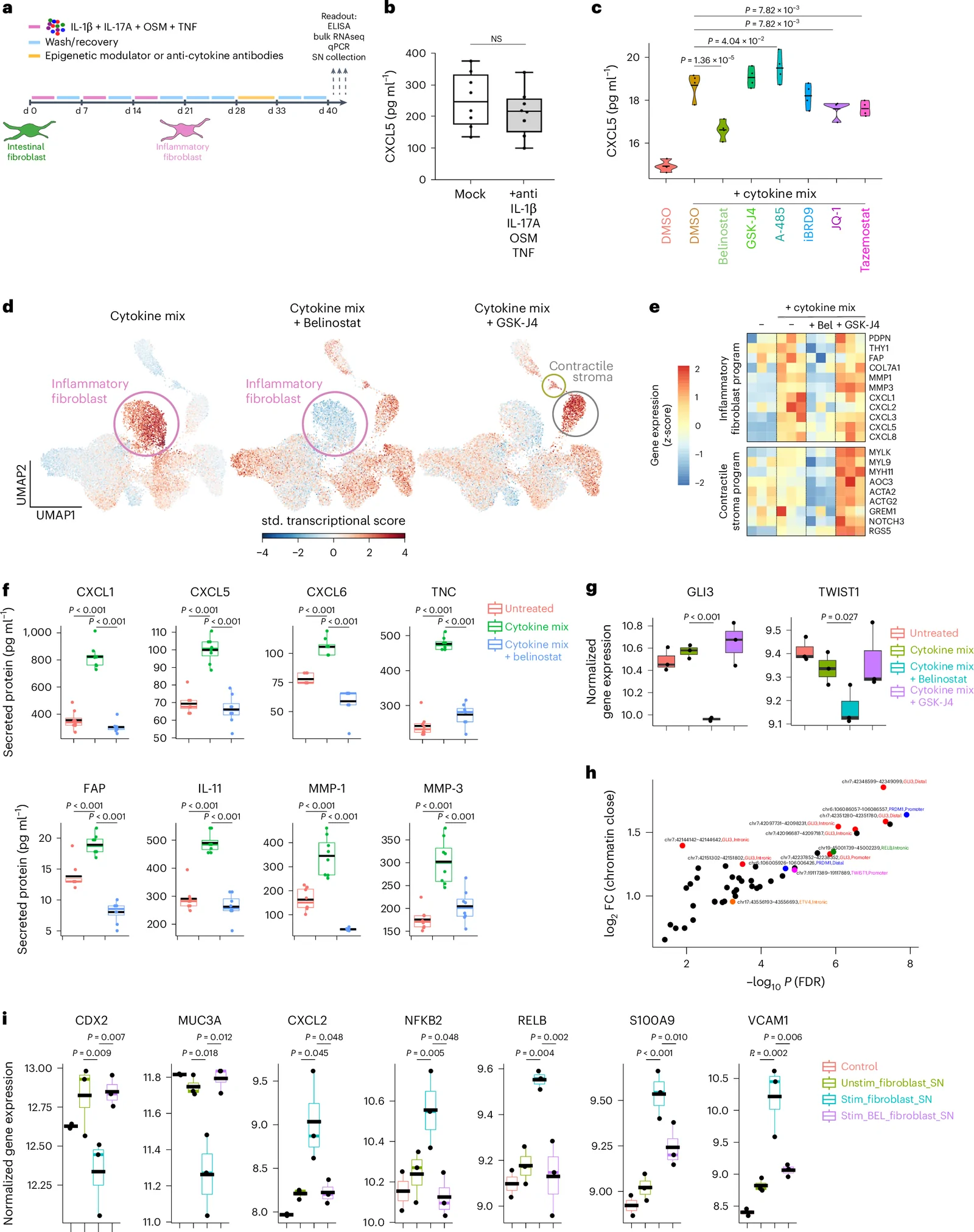
Epigenetic modulation mediates destabilization of the IF state which ameliorates mucosal inflammation. **a**, Schematic of the experimental approach to generate the inflammatory fibroblast (IF) phenotype in vitro; Ccd18-Co intestinal stromal cell line is treated with the combination (mix) of IL-1β (0.1 ng ml^−1^), IL-17A (10 ng ml^−1^), OSM (10 ng ml^−1^) and TNF (10 ng ml^−1^) (72 h each, followed by a 96 h recovery period by washing in complete culture medium), for three pulses in total. After the last pulse, cells were washed and left in recovery medium for at least 1 week before being exposed to vehicle control (dimethylsulfoxide (DMSO)) or epigenetic modifiers for 5 consecutive days. Following an additional 2 weeks of a recovery/wash phase, cell cultures were profiled by bulk RNA-seq (*n* = 3 replicates per treatment group), bulk ATAC-seq (*n* = 6 replicates per treatment group) or ELISA of culture supernatants. **b**, ELISA measurement of hCXCL5 secreted from in vitro generated IFs, either treated with a mix of anti-cytokine neutralizing antibodies or vehicle (PBS); *n* = 6 replicates per treatment group, *P* values derived from pairwise *t*-test. Boxplots span 25th to the 75th percentile with whiskers extending to the minimum and maximum; horizontal bars indicate the median. NS, not significant. **c**, ELISA measurement of hCXCL5 secreted from in vitro generated IFs, either treated with noncytotoxic concentrations of epigenetic modifiers (2,700 nM each, except for A-485 which was used at 540 nM) or vehicle-treated (DMSO 1:5,000 dilution) as in **a**); *n* = 4 replicates per treatment group, *P* values derived from post-hoc pairwise *t*-test FDR-corrected for multiple testing. Horizontal bars inside violins indicate the mean. **d**, UMAP representation of bulk RNA-seq signatures derived from in vitro generated treatment groups, as in **a**), scored on CD patient tissue-derived chromium suspension scRNA-seq stromal cell states. Only scores of upregulated transcripts are shown. **e**, Heatmap showing normalized gene expression of individual IF or CS marker transcripts across different treatment groups as in **d**); *n* = 3 replicates per treatment group. **f**, Luminex measurement of secreted protein (pg ml^−1^) from fibroblast cultures either left untreated or polarized into IFs by cytokine mix ± belinostat; *n* = 8 biological replicates per treatment group, FDR derived from pairwise two-sided *t*-tests with the reference group ‘Cytokine mix’. Boxplots span 25th to the 75th percentile with whiskers extending to 1.5× of IQRs; colored horizontal bars indicate the median, black horizontal bars indicate the mean. **g**, Variance stabilizing transformation normalized gene expression (bulk RNA-seq) of GLI3 and TWIST1 in the experimental setting shown in **a**); *n* = 3 biological replicates per treatment group, FDR values derived from pairwise two-sided *t*-tests with the reference group ‘cytokine mix’. Boxplots span 25th to the 75th percentile with whiskers extending to 1.5× of IQRs; colored horizontal bars indicate the median, black horizontal bars indicate the mean. **h**, IF were generated in vitro and treated with Belinostat as shown in Fig. 6a. 2 weeks after the last belinostat treatment, bulk ATAC-seq was conducted, and differential open chromatin analysis between IF (cytokine mix-treated) and IF + belinostat (cytokine mix + belinostat) was determined (Supplementary Table 35). Significant (FDR < 0.05, pairwise two-sided Wilcoxon test) changes for chromatin accessibility in GLI3, TWIST1, PRDM1, RELB and ETV4 loci are shown. The log_2_ fold changes (FCs) represent a reduction in chromatin accessibility (close). **i**, Variance stabilizing transformation normalized gene expression (bulk RNA-seq) of transcripts in the gut-on-a-chip after perturbation; *n* = 3 replicates per treatment group, *P* values derived from pairwise two-sided *t*-tests with the reference group ‘stim_fibroblast_SN’. Boxplots span 25th to the 75th percentile with whiskers extending to 1.5× of IQRs; colored horizontal bars indicate the median, black horizontal bars indicate the mean.

**Extended Data Fig. 8:**
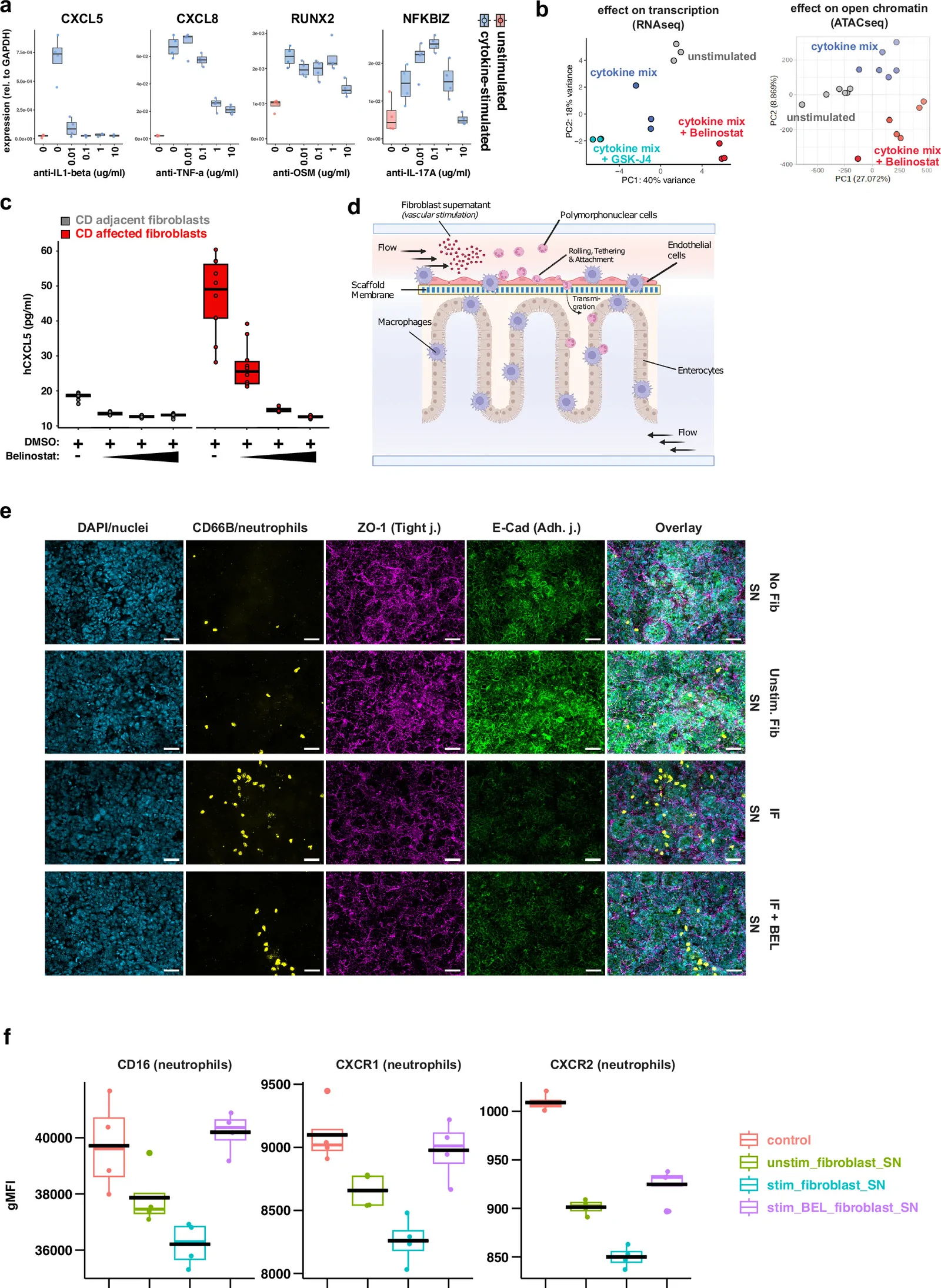
Epigenetic modifier effects and gut-on-a-chip model. **a**, Relative gene expression following single-cytokine stimulation in the presence of increasing concentrations of the indicated neutralizing antibody (0.01 µg/ml, 0.1 µg/ml, 1 µg/ml, 10 µg/ml). Each panel corresponds to the antibody specified in the plot title; n = 4 replicates per condition. Target transcript expression was assessed by qPCR, expression normalized to GAPDH is shown (2 ^ delta ct). Boxplots span 25^th^ to the 75^th^ percentile with whiskers extending to 1.5x of IQRs; horizontal bars indicate the median. **b**, Principal component analysis of the transcriptional and open chromatin profiles of Ccd18-Co human intestinal stromal cells 2 weeks after either polarization into IFs with cytokine mix alone, or after additional treatment with Belinostat or GSK-J4. Derived from bulk RNA-seq (n = 34 replicates per treatment group) or bulk ATAC-seq (n = 6 replicates per treatment group), respectively. **c**, ELISA measurement of hCXCL5 secreted from primary fibroblasts expanded either from affected or adjacent tissue of the same patient with CD, either treated with increasing concentrations (250 nM, 500 nM, 1uM) of belinostat or vehicle control (DMSO); n = 8 replicates per treatment group. Boxplots span 25^th^ to the 75^th^ percentile with whiskers extending to 1.5x of IQRs; horizontal bars indicate the median. **d**, Schematic of the experimental setup for the gut-on-a-chip experiment, following {Feile, 2024 #399}. Image(s) provided in part by Servier Medical Art (https://smart.servier.com) and BioRender.com, licensed under CC BY 4.0 (https://creativecommons.org/licenses/by/4.0/). **e**, Examples of cell markers and epithelial junction proteins detected by immunofluorescence in the gut-on-a-chip system, after the addition of control medium, unstimulated Ccd18-Co fibroblast supernatant, or stimulated IF-polarized fibroblast SN either treated or untreated with Belinostat; neutrophils were added to the circulatory system after 24 h pre-incubation with these supernatants. Images are representative of n = 3 replicate images. Scale bars: 50 um. **f**, Surface expression of chemotactic and Fcγ receptors in primary human neutrophils following 4 h incubation with Ccd-18Co fibroblast supernatants (as described in e). Neutrophils were obtained from healthy donor peripheral whole blood (n = 1) and were tested as n = 4 replicates per treatment group. Data are expressed as geometric Mean Fluorescence Intensity representing relative surface protein density measured by flow cytometry. Boxplots span 25^th^ to the 75^th^ percentile with whiskers extending to 1.5x of IQRs; black horizontal bars indicate the mean, colored horizontal bars indicate the median.

Given the observation that the cytokine-induced IF state does not spontaneously revert to a homeostatic fibroblast state, we asked which cues could destabilize it in vitro (Fig. 6a). We first ruled out that anti-cytokine therapy, as used in clinical practice (biologics), could reverse an established IF state, as neutralizing TNF, OSM, IL-1β and IL-17A (Extended Data Fig. 8a) did not alter the IF phenotype once polarized (Fig. 6b).

Epigenetic modifications like histone acetylation and methylation alter chromatin accessibility^35^, which may regulate the open chromatin configuration characteristic of the IF state. To this end, we chose a range of established small-molecule inhibitors representative of different classes of epigenetic modifier compounds, capable of remodeling or maintaining different chromatin states: class I histone deacetylase inhibitor (belinostat), histone acetylase inhibitor (A-485), H3K27 histone demethylase inhibitor (GSK-J4), the H3K27 methyltransferase inhibitor (tazemostat) and bromodomain inhibitors (iBRD9, JQ-1). We treated in vitro polarized IF with each of these compounds at noncytotoxic concentrations, and assessed the impact at least 2 weeks after the treatment (Fig. 6a). Notably, a HDAC inhibitor (belinostat) showed the most marked capability in reducing CXCL5 production from IF (Fig. 6c). IBRD9, JQ-1 and tazemostat showed significant, but smaller effects in the same direction (Fig. 6c). GSK-J4 had no effect on CXCL5 secretion from IF, so we used GSK-J4 as a negative control in subsequent experiments. Additional bulk ATAC-seq and RNA-seq revealed that both belinostat and GSK-J4 evoked substantial shifts in the transcriptional and open chromatin profiles of cytokine-polarized IF (Extended Data Fig. 8b). Projecting these transcriptional and epigenetic signatures (Supplementary Tables 31–35) back onto our suspension single-cell data, we confirmed that belinostat destabilized the IF phenotype (Fig. 6d,e), while not inducing cytotoxicity (Supplementary Fig. 2). Notably, GSK-J4 instead transformed IF into a state resembling fibrotic muscle (Fig. 6d), a CS state we had identified to predominate in areas of submucosal fibrosis.

We confirmed at a protein expression level that the modulation by belinostat was not restricted to CXCL5 but reflected an overarching reduction of functional proteins secreted from IF, such as CXC-type chemokines, TNC and FAP, IL-11 and MMP 1 and MMP3 (Fig. 6f). To further validate the physiological relevance of these findings made in a fibroblast cell line, we expanded primary fibroblasts from affected and adjacent tissue of a patient with CD. Fibroblasts isolated from the affected tissue secreted higher amounts of the IF marker CXCL5, which was inhibited by increasing concentrations of belinostat (Extended Data Fig. 8c).

Next, we asked how belinostat exerts its effects on IF, analyzing its modulation of IF TFs we had identified, and additional ones that were reported in literature recently. Belinostat significantly downregulated the transcript levels of TFs TWIST1 and GLI3 (Fig. 6g), which we had identified among the top loadings of open chromatin programs in IF (Fig. 3j). This was in line with a significant reduction in chromatin accessibility in *TWIST1*, *GLI3*, *ETV4*, *RELB* and *PRDM1* loci (Fig. 6h).

To further test the impact of belinostat on IF function, we utilized a human gut-on-a-chip system, replicating the epithelial-vascular-immune mucosal interface under circulating media flow mimicking blood vessels^36^. This model allowed to us measure neutrophil transmigration across the epithelial barrier, one of the effects supposed to be exerted by IFs due to their high expression and secretion of neutrophil chemoattractants (CXC-type chemokines). To this end, we collected supernatants from either unstimulated, IF-polarized or IF-polarized and belinostat-treated fibroblasts after the resting/wash period as depicted in Fig. 6a. We then stimulated the vascular side of the gut-on-a-chip system with these supernatants, and measured transcriptional changes in the system after introducing neutrophils to the flow (setup as in Extended Data Fig. 8d). The soluble factors contained in the supernatant (SN) of IF-polarized fibroblasts reduced the markers of epithelial barrier integrity (CDX2 and MUC3A), but increased the markers of NF-κB activation (NFKB2 and RELB) and neutrophil chemoattractants (CXCL3), as well as the markers of vascular activation (VCAM1); S100A9 expression was increased as expected due to the epithelial transmigration of neutrophils (Fig. 6h). Of note, if SN was derived from IFs treated with belinostat, the changes were reversed (Fig. 6h), which was confirmed by immunofluorescence staining (Extended Data Fig. 8e). Given the proximity of IFs to blood vessels in the ulcer bed, it is plausible that IFs are one of the first cell types encountered by extravasating neutrophils in the tissue. To test this, we incubated blood-derived human neutrophils with supernatants of fibroblasts. This demonstrated a direct effect of fibroblast supernatants on neutrophil activation, including the downregulation of CD16 and tissue homing receptors CXCR1 and CXCR2 on the surface of blood-derived neutrophils (Extended Data Fig. 8f); this effect was increased if supernatants from IF-polarized fibroblasts were used, but ameliorated when supernatants from belinostat-treated IFs were applied (Extended Data Fig. 8f).

Altogether, our experiments reveal that differential modulation of IFs in vitro with epigenetic modifier compounds can either destabilize the IF state or drive it toward the CS state. The destabilization of the IF state by belinostat prevents IF secretome-induced neutrophil transmigration across the epithelial barrier, and neutrophil activation. Therefore, differential epigenetic modulation can alter the stromal phenotypes that constitute pathological inflammatory and fibrotic tissue microniches.

## Discussion

Stromal cells have gained considerable attention for their association with chronic inflammatory disease and cancer^37^. In this study, direct in situ mapping through single-cell resolution spatial transcriptomics provided a refined, unbiased, and systematic annotation of intestinal stromal cell states in full-thickness CD bowel tissue. CD-associated changes in stromal cell state heterogeneity have been extensively mapped across human^11,13,15–19,21,22,38^ and mouse^39,40^, but so far it remained unknown how pathology-associated states are generated and how their phenotypes are stabilized by the tissue niches they reside in. We dissect the cellular environments of two stromal cell states (IFs and CS) that dominate in the microniches that can contribute to the pathological thickening of the bowel wall in CD: mucosal inflammation in the form of (fissuring) ULC and submucosal FIB. These findings resolve previous uncertainty as to whether IF-like stromal states are restricted to ulcerated regions that are adjacent to, but distinct from, fibrotic zones. In some patients we detected a TNC^hi^ myofibroblast phenotype in submucosal fibrotic areas directly adjacent to ulcers, displaying an intermediate phenotype between IF and contractile muscle. It is plausible that IFs differentiate from myofibroblasts, or vice versa, during simultaneous chronic inflammation and fibrotic remodeling of the gut in CD. Combinatorial cytokine stimulation downregulates programs associated with submucosal universal fibroblast precursor populations, previously shown to give rise to most tissue-resident fibroblasts across organs^34^. On our in silico trajectory from universal to inflammatory fibroblasts lies a population of submucosal myofibroblasts, but as for all single-cell trajectory analyses, these findings only suggest possible ontogeny relationships, which are to be experimentally validated.

Notably, recent findings report stromal cell states that strongly resemble IF (high expression of CXC-type chemokines, IL-11, MMPs 1/3 and CHI3L1) enriched in intestinal fistula tracts found in CD^19,21^. Our analysis (Extended Data Fig. 6e) demonstrates a high similarity of IF with the fistula-associated stroma described my McGregor et al. It is therefore tempting to speculate that IF in mucosal ulcers are the origin of deeper fissures, and eventually transmural fistula tracts, further highlighting their potential as therapeutic targets for difficult-to-treat CD. Similarly, IF-like phenotypes are found along the GI tract in different conditions, including pre-inflammation oral gingival mucosa (Extended Data Fig. 6f, g). We recently identified an ‘inflammatory myofibroblast’ population that shares features of (IL-11, MMP and CXCL expression) in early skin wounds, which shows similarities to fibroblast populations associated with several inflammatory diseases leading to scarring and cancer^41^. This evidence highlights the IF state as a potential shared phenotype across diseases and organs in chronic inflammation and pre-inflammatory niches^20^.

By chromatin accessibility profiling, we demonstrate that IF and CS transcriptional states are characterized by distinct open chromatin configurations. Through in silico regulon analysis and experimental validation, we reveal that IFs show strong enrichment in activity of several TFs. Of these, RNA-silencing experiments validated PRDM1, ETV4 and RELB to differentially impact on CXC-type chemokines and MMP versus collagen expression, key functional properties of IFs. Of note, another member of the ETS family TFs (*ETS2*) has been identified very recently to modulate inflammatory macrophage phenotypes^42^, highlighting the possibility of conserved inflammatory molecular hubs utilized by different cell types. Belinostat reduced the expression of GLI3 and TWIST1, resulting in a destabilization of the IF phenotype by downregulation of key markers such as CXC-type chemokines, MMPs and IL-11. Recently, *TWIST1* has been identified as a stabilizing TF for a motile fistula-associated fibroblast state in CD^21^. *GLI3* shares high homology with *GLIS3*, recently experimentally validated to promote an IF phenotype^22^. Belinostat reducing GLI3 and TWIST1 transcript expression and chromatin accessibility represents a viable pharmacological perturbation strategy for IFs. The relevance of our findings is further emphasized by previous reports on increased HDAC expression in active CD^43^.

This is in line with our findings revealing changes in open chromatin configuration as a hallmark of stromal states residing in pathologic tissue niches, and that repeated, combinatorial cytokines can mediate this change in the case of IF. Generating integrative spatial maps of stromal cytokine responses and immune environments across the CD-affected gut, we demonstrate that IFs localize to immune cell-rich ulcer regions with high cytokine signaling activity. We validated experimentally that combinatorial cytokine stimulation results in the generation of a stable IF phenotype that does not spontaneously revert. While we observed efficient induction of the IF phenotype with a combination of IL-1β, TNF, IL-17A and OSM, we cannot rule out that other cytokine combinations would have the same effect, activating convergent signaling pathways. Our reductionist in vitro setting may mirror the chronic inflammatory tissue environment in CD, where homeostatic stromal cell populations are repeatedly exposed to multiple inflammatory cytokines through the immune cells homing to inflamed tissue sites. Notably, CS residing in immune-depleted fibrotic areas did not show a cytokine responsiveness comparable to IFs. Once polarized by combinatorial cytokines, the IF phenotype does not revert upon withdrawal or blockade of the inducing cytokines, suggesting a stable phenotype and aligning with the distinct open chromatin profiles of IF we were able to isolate from CD tissues. Consequently, we demonstrate that epigenetic modulators can change the phenotype of IFs and functional properties. This effect is not broadly achieved by any epigenetic modulator, but most prominently by belinostat, a class I HDAC inhibitor^44^ By contrast, applying a H3K27 demethylase inhibitor, GSK-J4 (ref. ^45^) promoted CS-like programs in treated fibroblasts. As discussed above, GREM1^hi^ fibrotic muscle and TNC^hi^ myofibroblasts were found to be the major patient-dependent stromal population in fibrotic lesions in CD. We hypothesize that the muscle cells or myofibroblasts in the fibrotic region reflects either (1) different stages in the fibrotic remodeling or (2) stage-independent expansion of either muscle or myofibroblast that outcompete other cell types in this niche.

These results considerably expand the view and possible utility of HDAC inhibition and clarifies a potential target cell population in IBD^46,47^. The use of epigenetic modulation is a topic of growing interest across several diseases^48^, including oncology, where such agents are already in clinical use. Nonetheless, their known toxicity profiles may be more acceptable in cancer than in chronic inflammation (CD). Irrespectively, our findings provide a new conceptual framework of pathologic tissue microenvironments evoking stromal states that display unique open chromatin configuration, induced by multi-cytokine hits in the case of IF.

Our findings have potential future implications for the clinical management of refractory CD, where a frequent last resort is the surgical resection of affected bowel. While adopting an aggressive top-down therapy in CD will likely reduce the rates of surgery in the future^49^, our findings suggest a framework for therapy resistance observed beyond a point where current medications remain efficacious: once IFs are established via chronic inflammation, their chromatin profile has also changed and anti-cytokine therapy becomes less efficacious. Epigenetic modifiers or derived assets could destabilize the IF phenotype offering a new therapeutic angle.

Overall, our findings establish the concept of how maladapted pathologic tissue niches including cytokine networks shape distinct stromal states with characteristic open chromatin profiles. This offers a new conceptual approach to further our understanding of pathogenic processes in CD. In addition, the spatial and open chromatin atlas of stromal cells in CD, stromal cytokine response maps, and the development of a robust in vitro model of IFs add valuable resources to this field of study.

## Methods

### Statistics and reproducibility

For cohort-level analyses and profiling, no statistical method was used to predetermine sample size, patients were not randomized and data collection and analysis were not performed blinded to the conditions of the experiments. Data distribution was assumed to be either normally or not-normally distributed based on the origin of the data (for example, patient samples and in vitro experiments) but was not formally tested. Cell line-based in vitro experiments were successfully independently repeated at least once. Depending on the data distribution and type, an appropriate statistical test was applied as indicated in figure legends and tables. Generally, experiments were only included in the final analysis if internal controls were valid. Individual data points were excluded if they represented clear outliers from the data distribution together with a plausible technical reason.

### Patient cohorts and ethics

There was no defined recruitment strategy for this study. Samples were picked and analyzed retrospectively. Patients previously diagnosed with CD were recruited from the IBD clinic at the John Radcliffe Hospital or the Churchill Hospital in Oxford. Tissues were collected at surgery or from archives (formalin-fixed paraffin-embedded; FFPE) under research ethics protocols 09/H1204/30, 16/YH/0247 or 21/YH/0206. Written informed consent was obtained from all participants before the collection of any data or samples.

### Histopathology and FFPE tissue sectioning

Tissues were obtained by dissection from surgical specimens and fixed in in 4% methanol-free paraformaldehyde for 24 h, followed by dehydration in an ethanol gradient and xylene, then embedding in paraffin blocks (FFPE). The 5-µm sections were then sectioned and stained (H&E using standard protocols). Whole-section imaging of H&E sections was performed on a NanoZoomer digital slide scanner (Hamamatsu). Alternatively, 5-µm-thick tissue slices were sectioned onto Xenium slides for profiling.

### 10x Xenium spatial transcriptomics

The 5-μm FFPE tissue sections were prepared and processed according to the manufacturer’s guidelines for profiling with Xenium Prime 5k Human Pan Tissue & Pathways Panel, or Xenium Human MultiTissue & Cancer Panel with a custom add-on panel. For segmenting the Xenium Prime 5k data, Xenium MultiTissue Stain Mix was used with three distinct methods selected by a deep-learning model: prioritized according to boundary stain (ATP1A1^+^CD45^+^E-cadherin), interior stain (18S) and nucleus expansion at a 5.0-µm radius. Segmented cells were filtered requiring ≥16 probe counts and ≥20 genes detected. For the remaining cells across the four samples, the median probe count was 146.0 and the median unique genes per cell was 128.0; the extended *n* = 12 samples had comparable median probe counts, which for all *n* = 16 samples ranged 84–294 median cell counts per sample and 74–237 median unique gene cell counts per sample. Counts were normalized by cell to 10,000 in total and pseudo log transformed. Separate integration and annotation analyses were carried out on the *n* = 4 and combined *n* = 16 datasets (Extended Data Fig. 1a). To assist in cell type annotation, cell type labels were predicted with CellTypist (v.1.6.3) models trained on a subset of the pan-GI atlas and the scRNA-seq of fibroblasts from this study^17^.

### Spatial transcriptomics cell annotation

To assist in cell type annotation, cell type labels were predicted with CellTypist (v.1.6.3) models trained on a subset of the pan-GI atlas, consisting of cells from small intestine healthy and disease samples, the scRNA-seq of fibroblasts from this study, and recursively from first-pass annotation of *n* = 4 Xenium 5k data transferring labels to the extended *n* = 16 dataset (Extended Data Fig. 1a). CellTypist was trained without feature selection enabled optimizing the overlap with the Xenium probe set. The maximum probability CellTypist prediction was used per cell, denoising by majority voting among Leiden clusters at a resolution parameter of 30, while requiring a prediction confidence >0.3. To integrate across the four samples, scVI (v.1.2.0) was used with principal-component analysis (PCA) dimensionality reduction and 2,000 highly variable genes. Variational autoencoder scVI models were trained assuming negative binomial likelihood with two hidden layers and 30 latent variables. The integrated data were visualized using 15-nearest-neighbor graphs and UMAP with default parameters in scanpy (v.1.9.6). Based on inspection of integrated cell embeddings and markers (two-tailed Wilcoxon test, one-versus-rest, Benjamini–Hochberg (BH) correction), predicted annotations with <200 cells were excluded as where CellTypist predictions for cells with <100 probes, which otherwise were biased toward enterocyte false-positive predictions. Throughput, spatial plots were visualized with squidpy (v.1.6.0) unless otherwise stated.

To annotate the mesenchymal compartment of the Xenium 5k data in the *n* = 4 dataset, broad lineage predictions (CellTypist) from the pan-GI atlas were subset to ‘mesenchymal’ cells, excluding 17% of cells with non-mesenchymal fine annotation, leading to data on 181,597 stromal cells. These cells were subclustered based on the global scVI latent embedding at Leiden resolution 5.0. The clusters were manually annotated by comparing fine-grained CellTypist predictions based on the pan-GI atlas with predictions from CellTypist models trained on the fibroblast suspension scRNA-seq presented in this study, along with identified marker genes (two-tailed Wilcoxon test, one-versus-rest, BH correction). The fibroblast annotations were subsequently manually curated, merging subclusters of smooth muscle cells (SMCs) along with smaller clusters mapping to identical scRNA-seq subtypes. The resulting fibroblast annotations were renamed based on expert knowledge of fibroblast function in the gut, considering the observed spatial localization of the fibroblast subtypes. Similarly, cells in the T cell compartment were clustered and annotated based on established subtype marker genes. To transfer labels to the full *n* = 16 Xenium 5k data, including mesenchymal and T cell annotations, we trained CellTypist models on the first-pass annotations and applied them to *n* = 16 data, requiring confidence >0.3. Moreover, by identifying mismatches between broad lineage predictions from pan-GI models and predicted fine-level annotation, we were able to filter out low-quality predictions including numerous low-quality enterocyte predictions. However, several of such cases also reflected adjacent cells presumably due to a mixture of spillover effects, annotation artifacts and true biological signal. Invariably such cells reflected cells known to closely interact, which we in the annotation designated as adjacent cell types rather than excluded as artifacts. We investigated each lineage compartment separately using scVI sub-embeddings, taking into account the CellTypist confidence scores and predictions as well as known markers genes, identifying that annotation revisions were necessary for myeloid, epithelial, endothelial, B cell but not the mesenchymal or neural compartment. Using Leiden clustering at varying resolutions, we were able to validate predictions and define additional cell types such as: T cell subtypes, MUC5B goblet, Epithelial_stem, B_germinal center, endothelial cells subdivided by arterial, venous and capillary. We also found macrophage subtypes: B cell-adjacent, subepithelial, CD14^hi^, SMC-associated, CXCL12, neuron-associated, phagocytic, CXCL9, in agreement with the functional diversification of intestinal macrophages^50^.

### CellCharter cellular niche identification

To infer cellular niches CellCharter (v.0.3.2) was used. Neighborhood graphs were calculated by Delaunay triangulation and long edges removed at a 99 percentile cutoff. Next, integrated scVI embeddings were aggregated at a depth of three layers of the neighborhood graph. Spatial Gaussian mixture models were tested at a range spanning 4–25 clusters, evaluating cluster stability with the Fowlkes-Mallows index as previously described (https://www.nature.com/articles/s41588-023-01588-4). After picking an optimal number of spatial clusters, enrichment of cell types was computed by niche using a permutation test (*M* = 1,000, log_2_FC) implemented in the CellCharter package.

### CatsCradle spatial ligand–receptor analysis

Spatial ligand–receptor interactions on ULC tissue niches were inferred via CatsCradle (v.1.4.2) (https://github.com/AnnaLaddach/CatsCradle) applied to Xenium spatial transcriptomics data. Neighborhood graphs were constructed per sample using Delaunay triangulation, and edges longer than 100 µm were removed. Neighborhoods were extended to the fourth degree and collapsed to generate aggregated spatial adjacency graphs. Ligand–receptor analysis was performed for the ULC niche using the analytical method implemented in CatsCradle (human database, minEdgesPos = 10, conditional = FALSE). *P* values were adjusted using the BH method, and significant interactions were defined as FDR < 0.05 with a mean interaction score >0.01.

### Isolation of cells from human intestinal tissue

Surgical specimens were cleaned up by dissection, and the external muscle layer removed after whole-thickness samples were taken for pathology/spatial work. Remaining mucosa and submucosa were minced using surgical scissors and scalpel blades, after removal of bulk epithelial cells by two washes in PBS containing antibiotics (penicillin–streptomycin, amphotericin B, gentamicin and ciprofloxacin) and 5 mM EDTA (Sigma-Aldrich). Minced tissue was then subjected to multiple rounds of digestion in RPMI 1640 medium, 5 mM HEPES, antibiotics as listed above and 1 mg ml^−1^ collagenase A and DNase I (Sigma-Aldrich). After 30 min, digestion supernatant containing cells was removed, filtered through a cell strainer, spun down and resuspended in 10 ml of PBS containing 5% bovine serum albumin (BSA) and 5 mM EDTA. The remaining tissue was then topped up with fresh digestion medium until no more cells were liberated.

### Fluorescence-activated cell sorting

Single-cell suspensions obtained from tissue digests were blocked in PBS 5% BSA containing human anti-Fc block (Miltenyi) stained for FACS analysis or sorting with Fixable Viability Dye eFluor 780 (eBioscience, 1:1,000 dilution), DAPI (Invitrogen, 1:50,000 dilution, H3570) and antibodies (all 1:200 dilution; FITC anti-human EPCAM (BioLegend, 324204, clone #9C4); BV510 anti-human CD90 (BioLegend, 328126, clone 5E10); AF700 anti-human CD45 (BioLegend, 304024, clone #HI30); APC anti-human PDPN (eBioscience, 17-9381-42, clone #NZ-1.3); and BV605 anti-human CD31 (BioLegend, 303122, clone #WM59)) in PBS with 5% BSA and 5 mM EDTA for 20 min on ice. After washing in the same buffer, cells were sorted on a BD Aria III (100-µm nozzle) for the CD31^−^EPCAM^−^CD45^−^ stromal fraction either positive for CD90 or PDPN, or both. Sorted fractions were immediately processed for 10x single-cell/nuclei analysis by scRNA-seq or scATAC-seq and libraries were constructed as per the manufacturer’s instructions (10x Genomics). Both pooled RNA and ATAC libraries were sequenced (3′ paired end) on a NovaSeq6000 (28/98) and NextSeq (50/80/16/50), respectively.

### 10x scRNA-seq processing, quality control and annotation

Sequences were aligned to GRCh38 with CellRanger using STAR. scRNA-seq doublets were identified with Scrublet and excluded if *P* < 0.5 (BH-corrected) or Scrublet score > 0.3. Ribosomal, mitochondrial and ‘hb’ genes were excluded by string matching. For cell quality control (QC), standard metrics were calculated for: read counts, number of unique genes, percent top-50, along with percentages of mitochondrial, ribosomal and hb reads per cell. To threshold each QC metric, outliers were detected by fitting univariate ten-component Gaussian mixture models to each observed distribution, using pseudo log-transformed values for count data. Cells were excluded requiring >60% of cells to pass according to the auto_qc_filter() function in the scanpy_scripts package. Altogether, cell QC removed 7,567 of 45,683 cells.

Next, the cell by gene count matrix was normalized to 10,000 total counts and pseudo log transformed. To reduce dimensionality, PCA was calculated for 20 components based on the 3,000 most highly variable genes. In the reduced PCA space, a 15-nearest-neighbor graph was constructed. Samples were integrated using BBKNN (https://academic.oup.com/bioinformatics/article/36/3/964/5545955), trimming the number of neighboring cells to 50 at most. To visualize data for cell annotation, UMAPs were constructed for the adjusted neighbor graph with default parameters in scanpy.

To annotate the cells, a logistic regression classifier was trained on a 5% subsample of the Smillie et al. dataset^14^, including only the lamina propria and epithelial fractions. Predicted cell type labels were compared to Leiden clusters at multiple resolutions (0.1, 0.3, 0.5 and 0.5) picking 0.5. Based on visual inspection, some (3 out of 14) larger clusters were subclustered for finer annotation. Final clusters were assigned to predicted cell type by majority voting. Last, vascular endothelial cells were excluded from subsequent analyzes and UMAPs was recomputed for the subset neighborhood graph with default parameters in scanpy. Mesenchymal markers were identified with two-sided *t*-tests (BH-corrected) per filtered gene for each cluster versus the rest with the rank_genes_groups scanpy function.

### scATAC-seq processing

Processing of scATAC-seq data was performed from 10x CellRanger Arrow files with the default ArchR pipeline. Doublet scores were computed with the number of neighboring cell criteria set at *k* = 10 with the ‘UMAP’ method and ‘tf-logidf’ term frequency-inverse document frequency (TF-IDF) normalization. Doublet cells were excluded by the filterDoublet function with parameters cutEnrich = 1 and filterRatio = 1, in addition to requiring DoubletScore ≤ 30. To reduce dimensionality of the tile matrix data (500-bp bins), iterative latent semantic indexing (LSI) was performed followed by Harmony batch correction by sample. With the batch-corrected LSI representation, clustering of cells was conducted with a modularity-based graph clustering algorithm implemented in Seurat FindClusters function at resolution = 0.8. To predict scATAC-seq cell-type labels through label transfer, constrained integration with scRNA-seq data was carried out with the addGeneIntegrationMatrix function on the gene score matrix and the batch-corrected LSI embedding. Clusters in the scATAC-seq data were annotated by majority voting among the predicted cell type labels. Additional cell-based QC was carried out setting thresholds by manually inspecting distributions of percent_top50, TSSEnrichment, ReadsInBlacklist, PromoterRatio and NucleosomeRatio.

To generate a corresponding peak matrix, genomic peaks were called with MACS2 and merged into a reproducible peak set with default parameters in ArchR. Dimensionality reduction of the peak count data was carried out in scanpy as for the scRNA-seq data, including BBKNN batch correction but using 5,000 highly variable peaks.

### Multimodal integration and gene regulatory network inference

scATAC-seq and scRNA-seq data were integrated by stromal cell state using MultiMAP (v.0.0.1)^29^ on PCA for RNA and peak-based iterative LSI for ATAC. The resulting integrated MultiMAP embeddings were inspected for mixture of modality with UMAP visualization. The joint RNA and ATAC cells were then aggregated into metacells using SEACells v.0.3.3 initializing the number of metacells to an expected average of 75 cells per metacell with the number of waypoint eigenvalues set to 10. Unbalanced SEACell metacells with less than 10% mixture of either RNA or ATAC cells were excluded (<90% purity cutoff). We validated MultiMAP on a global integration task of the fibroblast subtypes using PCA for both RNA and ATAC data. To infer enhancers and gene regulatory networks, we used the SCENIC+ (v.1.0.0) pipeline^31^ to predict TFs and putative target genes as well as regulatory genomic regions with TF binding sites. CisTopic (pycistopic v.1.0.2) was applied to identify region topics and differentially accessible regions from the fragment counts as candidate regions for TF binding. CisTarget (pycistarget v.1.0.2) was then run to scan the regions for TF-binding sites, and GRNBoost2 (arboreto v.0.1.6) was used to link TFs and regions to target genes based on coexpression or accessibility. Enriched TF motifs in the regions linked to target genes were used to construct TF–region and TF–gene regulons. Finally, regulon activity scores were computed with AUCell based on target gene expression and target region accessibility, and regulon specificity scores (RSS) derived from them within the SCENIC+ framework. Standard filtering of regulons was applied and further filtered by TF cistrome correlation at a |rho| > 0.4 threshold.

### Trajectory analysis

Spliced versus unspliced ratios were computed with RNA velocity (scvelo v.0.3.2). Genes were filtered required minimum of 20 counts retaining 2,000 highly variable genes. First- and second-order moments were computed per cell based on 30 neighbors and 30 principal components. RNA velocities were estimated with the stochastic, dynamic, and deterministic approach, picking the latter due to greater qualitative concordance with trajectories in mouse fibroblast atlas^34^. To predict transition between fibroblast states with the principal of manifold connectivity, PAGA^33^ implemented in scanpy (v.1.9.6) was applied to the batch-corrected nearest-neighbor graph. To compute pseudotime for potential trajectories, high connectivity paths between selected fibroblast states were selected, followed by trajectory analysis with scFates (v.1.0.8)^51^ based on PCA embedding with the ‘ppt’ method for 15 coarse nodes and λ = 100, σ = 0.025 and nsteps = 200. The trajectory tree was rooted based on CD34 expression and CytoTRACE^52^ analysis measuring transcriptional diversity. Genes associated with pseudotime were tested with cubic spline regression with the mgcv R package called from scFates with default parameters (including BH correction for multiple hypotheses). Trajectory results were visualized on a UMAP of the subset of the nearest-neighbor graph.

### Single-cell gene programs

To decompose scRNA-seq count matrices into gene programs, consensus non-negative matrix factorization (cNMF) v.1.3.4 was used with 2,000 highly variable genes at 100 iterations^27^. The optimal number of factors (*k* = 17) was determined as a tradeoff between clustering stability (silhouette score) and reconstruction error, testing *k* ranging 4–25. Consensus programs were obtained by filtering outliers of cNMF run replicates at a density threshold of 0.2 (mean distance to *k*-nearest neighbors), removing 5% of replicates. Gene set enrichment analysis for top 100 genes per program was carried out with PANTHER 19.0 overrepresentation test (Fisher’s exact test, BH correction) for Gene Ontology biological process terms.

For the scATAC-seq programs, to infer single-cell programs from the chromatin accessibility peak matrix, the cNMF algorithm was reimplemented (_utils.run_cNMF function in https://github.com/Teichlab/sctk, v.0.2.0) and run with default parameters: consensus filtering density threshold of 2.0 and 100 iterations. Out of the total peak set, 3% (12,098) were used as highly variable features requiring a minimum mean peak count of 0.0002. As a measure of program stability, silhouette scores of *k*-means clusters (as used for scRNA-seq) led to selecting the *k* = 14 solution.

### Ccd18-Co culture and primary fibroblast isolation

The Ccd-18Co colon fibroblast cell line was obtained from ATCC (CRL-1459) and maintained up to passage 20. Primary fibroblast cultures were obtained by plating tissue cell isolates as described above in tissue-culture treated plastic vessels. After 24 h, non-adherent cells were washed off, and the adherent fraction containing fibroblasts expanded and used between passages 5 and 15. Both Ccd-18Co and primary fibroblasts were maintained and expanded and maintained in full growth medium (MEMα Sigma, 12571063), penicillin–streptomycin (Thermo, 15140148) and 20% FBS (Thermo, A5209501). For experiments, fibroblast cultures were seeded at 25,000 cells in 200 μl full growth medium in 48-well format tissue-culture plates, unless stated otherwise.

### Stromal cytokine response atlas generation

For bulk RNA-seq readout, 25,000 Ccd-18Co cells in 200 μl full growth medium were plated in 48-well format tissue-culture plates. For bulk ATAC-seq readouts, 200,000 Ccd-18Co cells in 1 ml full growth medium were plated in six-well tissue-culture plates. Following attachment, cells were starved in serum-free medium for 72 h, followed by stimulation with 0.1 ng ml^−1^ human recombinant IL-1b, or 10 ng ml^−1^ each of human recombinant IL-6, IL-17A, IL-22, IFN-a, IFN-g, OSM, TNF or TGF-β1 (all cytokines from Peprotech) in serum-free medium. After 24 h, cells were processed for RNA or open chromatin isolation.

### Bulk isolation of RNA and RNA-seq processing from cell populations

Isolates from cell cultures were prepared by direct lysis in RNA lysis buffer, followed by RNA isolation with the RNA was isolated using the QIAGEN RNeasy Mini kit (QIAGEN, 74104) or Zymo Quick-RNA 96 kits (ZymoResearch, R-1052) with the on-column DNA digestion step. Isolated RNA was stored at –80 °C until further processing or shipped to service providers. Library preparation and RNA-seq was conducted by service providers (Novogene, Azenta) with technical details indicated in the data deposits. Resulting. fastq raw files were quality checked using FASTQC v.0.11.9 (http://www.bioinformatics.babraham.ac.uk/projects/fastqc/) and MULTIQC v.1.14. (https://github.com/MultiQC/MultiQC), before quantification using KALLISTO (https://github.com/pachterlab/kallisto) v.0.48.0 with default settings. Processing of raw files was run in a Python virtual (v.3.10.8) environment using CGATcore (v.12.2.0) pipeline framework (10.12688/f1000research.18674.2).

### Differential expression and scoring in single-cell data

Differential expression was computed with negative binomial tests (DESeq2). Up- and downregulated genes (FDR < 0.05, BH, |log_2_FC| > 1.5, max top 1,000 genes) were scored in single-cell data using average normalized expression compared to random gene sets of matching size using the scanpy score_genes function. The scores were standardized by cell for visualization purposes.

### IF gene set fibroblast scoring and spatial distance analysis

To study the effect of spatial distance to the ULC niche on the fibroblasts’ transcriptomes, per-cell nearest-neighbor distances were computed using centroid coordinates extracted from Xenium annotated cells. For each affected sample and each cell, Euclidean distances to the nearest ULC cell were calculated using the phenoptr v.0.3.2 package and stored as metadata. An IF gene signature was derived from scRNA-seq differential expression (adjusted *P* < 0.05, avg_log_2_FC > 1.5). Following, Xenium data normalization, module scores were computed using AddModuleScore (ctrl = 100, seed = 13) based on the IF signature gene set. Finally, fibroblast subtype densities relative to the distance to ULC were estimated using kernel density functions per sample and summarized as mean ± s.e. Changes in IF gene-set expression as a function of distance were modeled using LOESS smoothing and visualized alongside fibroblast density distributions.

### *k*NN-based treatment prediction (bulk-to-single-cell prediction)

We used a *k*-nearest-neighbor (*k*NN)-based classification approach to assess the transcriptional similarity between our in vitro stromal model and the stromal populations identified in the scRNA-seq dataset. In brief, each cell from the scRNA-seq dataset is assigned to either the unstimulated or cytokine-stimulated condition based on the similarity of its transcriptomic profile to bulk RNA-seq profiles derived from untreated or cytokine mix-treated Ccd18-Co cells. To infer in vitro treatment states in stromal scRNA-seq data, TPM expression from cytokine treated and untreated fibroblasts were log_1_p transformed and technical replicates were collapsed by averaging. The resulting expression matrix was converted to a sparse matrix and used as reference in SCP v.0.5.6. Stromal scRNA-seq data were normalized and *k*NN-based predictions were computed using RunKNNPredict with shared genes between datasets. Low-confidence predictions were removed (similarity > 0.3). Predicted treatment labels were visualized on UMAP embeddings, and cluster-level composition was summarized using pie charts positioned at subtype centroids. Similarity was computed using cosine distance in a gene expression space, based on all genes shared between the bulk and single-cell datasets, and using *k* = 30 nearest neighbors. Cells with a cosine similarity score below 0.3 to their nearest bulk neighbor were excluded as low-confidence assignments. In Fig. 4h, cells are colored according to their predicted class (blue indicates unstimulated Ccd18-Co; red indicates cytokine-stimulated Ccd18-Co; and NA indicates low-confidence/unreliable prediction). For each cluster, pie charts represent the proportion of cells assigned to each condition, providing a summary of the mapping at the cluster level.

### scRNA-seq UMAP projections

To compare stromal populations across datasets, mesenchymal stromal cells were subset from full resection scRNA-seq data^21^, and projected onto the previously annotated stromal reference using SCP (v.0.5.6). Mapping was performed using the RunKNNMap function, using all genes shared between the query and reference datasets. Following pairwise distance calculation between query and reference cells in the common gene expression space, the 30 nearest neighbors (*k* = 30) in the reference dataset were identified for each query cell and used to calculate a UMAP coordinate based on their centroid. Finally, projections were visualized on the reference UMAP to assess correspondence between stromal subtypes.

### Bulk isolation of accessible open chromatin and ATAC-seq

For chromatin accessibility profiling of bulk cell populations (Ccd18-Co intestinal fibroblasts), the Omni-ATAC protocol was followed for nuclei isolation and library preparation^53^ was followed. In brief, fibroblasts were collected by trypsin-based detachment and pelleted by centrifugation. After confirming viability of >95% (Trypan blue hemocytometer), nuclei were isolated by incubating cells for 3 min on ice in nuclei isolation buffer containing 0.1% NP-40, 0.1% Tween20 and 0.01% digitonin. Following washes, nuclei were transposed (Illumina Nextera Tn5 transposase contained in FC-121-1030) for 30 min at 37 °C with 1,000 rpm mixing. Transposed DNA fragments were isolated using QIAGEN MinElute PCR Purification kit (28004) and pre-amplified for five cycles using NEBNext High Fidelity 2x PCR Master Mix (M0541) and Illumina Nextera primers (FC-121-1030). At this stage, the additionally required amplification cycles were determined by SYBR green (Invitrogen, S7563) based quantitative PCR. After amplification for additional cycles (between 3–6), amplified DNA was cleaned up using QIAGEN MinElute kit and quantified using KAPA absolute quantification kit (Roche, 07960166001). Resulting libraries were quality-controlled on an Agilent TapeStation 4200 using High Sensitivity DNA Screen Tape analysis D1000 (Agilent, 5067-5584). Libraries were then pooled and size selected using Ampure XP beads (Beckman, A63880), before re-quantification by TapeStation and adjustment to 2.8 pM and sequencing on an Illumina NextSeq500 or NovaSeq6000.

### Processing of bulk ATAC-seq data

Paired-end ATAC-seq data were processed with the ENCODE ATAC-seq pipeline v.2.2.3 (https://github.com/ENCODE-DCC/atac-seq-pipeline), including adaptor trimming, alignment to hg38, with cross-correlation analysis enabled, and peak calling with MACS2. Based on the csaw_workflow (https://github.com/reskejak/ATAC-seq), consensus peak sets were assembled across condition with GRanges from narrowPeak files filtered for 300,000 peaks and *P* < 0.01. Peaks were then counted using csaw v.1.32.0 with maximum fragment length of 1,000 excluding peaks overlapping with blacklist_hg38_unified, resulting in 334,857 consensus peaks across 18 samples and three conditions. Peaks were further filtered for low abundance at log_2_CPM > −3. Normalization factors for background correction at 10,000-bp bins were computed with the trimmed mean method. Adjacent peaks were then merged within 500 bp up to a maximum of 5-kb consecutive peaks. The resulting peaks were annotated with ChIPseeker v.1.34.1 according to the TxDb.Hsapiens.UCSC.hg38.knownGene genome database.

To analyze differential accessibility, negative binomial models were fitted with edgeR v.3.40.2 using the glmQLFit function. To analyze TF enrichment in differentially accessible peaks, pycistarget v.1.0.3 was used with a custom motif database generated using the v10nr_clust_public motif collection from the Aerts laboratory (https://resources.aertslab.org/cistarget/motif_collections/v10nr_clust_public). For each condition, we filtered differentially accessible peaks for adjusted *P* value < 0.01 and logFC ≥ 0.5 and used the function run_cistarget() to detect significantly enriched motifs with a normalized enrichment score (NES) ≥ 3. Both direct and orthology-based annotations were used to match motifs to TFs.

### Scoring of bulk open chromatin in single-cell data

To map the variable length peak sets from bulk ATAC-seq (MACS2 calls) to the 500-bp fixed-width peaks identified with ArchR in the single-cell ATAC-seq data, overlapping genomic coordinates were required to be >300 bp. Matched differentially accessible peaks from bulk ATAC-seq data were scored in the single-cell data by computing the average normalized peak counts compared to (subtraction) randomized control peaks using the scanpy score_genes function. Down- and upregulated peaks were scored separately requiring FDR < 0.05 (BH) for at most 5,000 top-ranking peaks per comparison.

### In vitro inflammatory fibroblast model

Ccd-18Co cells were seeded at 25,000 cells in 200 μl of full growth medium in 48-well tissue-culture plates. After attachment, the Ccd18-Co intestinal stromal cell line was treated with the combination (mix) of IL-1b (0.01 ng ml^−1^), IL-17A (10 ng ml^−1^), OSM (10 ng ml^−1^) and TNF (10 ng ml^−1^) (72 h each, followed by 96 h of a recovery period by washing in complete culture medium), for three pulses in total. After the last pulse, cells were washed and left in recovery medium for at least 2 weeks profiling by bulk RNA-seq (*n* = 3 replicates per treatment group), bulk ATAC-seq (*n* = 6 replicates per treatment group) or ELISA of culture supernatants.

### Epigenetic modulator perturbations

The Ccd18-Co intestinal stromal cell line was treated with the combination (mix) of IL-1b (0.01 ng ml^−1^), IL-17A (10 ng ml^−1^), OSM (10 ng ml^−1^) and TNF (10 ng ml^−1^) (72 h each, followed by 96 h of a recovery period by washing in complete culture medium), for three pulses in total. After the last pulse, cells were washed and left in recovery medium for at least 1 week before being exposed to vehicle control (DMSO) or epigenetic modifiers for five consecutive days. After 5 days, cells were returned to full growth medium for a recovery period of at least 2 weeks; during recovery, the medium was changed four times to remove any potential traces of epigenetic modulator compound. Supernatants were collected after this period, or cells were processed for readouts as indicated. For all modulator treatments, the final concentration of vehicle (DMSO) was kept constant at 1:5,000 dilution in all treatment groups.

### Cytokine neutralization and quantitative real-time PCR

Single-cytokine stimulation solutions (TNF, OSM, IL-17A and IL-1β) were prepared in serum-free (starvation) medium and incubated with their corresponding neutralizing antibodies (anti-OSM, mouse monoclonal #17001, MAB295 (R&D), 10 µg ml^−1^; anti-IL-1β, #80516, MAB201 (R&D), 10 µg ml^−1^; anti-TNF, mouse monoclonal #28401, MAB610 (R&D), 10 µg ml^−1^; and anti-IL-17A, mouse monoclonal #41809, MAB317 (R&D), 10 µg ml^−1^). Cytokine–antibody mixtures were incubated for 2 h at room temperature before being applied to 48-well tissue-culture plates seeded with 50,000 Ccd-18Co cells. Cells were treated with 200 µl per well of either cytokine–antibody mixture, vehicle control or untreated control medium. After 3 h of exposure, cells were lysed, total RNA was isolated, and complementary DNA was generated by reverse transcription. Cytokine neutralization was validated by qPCR using previously assessed TaqMan Gene Expression Assays from Thermo Fisher Scientific (CXCL5 (Hs01099660_g1), CXCL8 (Hs00174103_m1), RUNX2 (Hs01047973_m1) and NFKBIZ (Hs00230071_m1).

### WST-8 assay

Cell viability was assessed using a colorimetric, tetrazolium reduction-based assay (Cell Counting Kit 8/WST-8 / CCK-8, Abcam, ab228554). Cells were incubated with WST-8 reagent diluted 1:20 in culture medium at 37 °C for 30 min after which the reaction solution was transferred to a flat-bottom 96-well plate and absorbance was measured at 460 nm using a microplate reader. Raw absorbance values were blank corrected using wells containing only assay medium.

### Brightfield imaging

Brightfield images of adherent fibroblasts were acquired using a ZEISS Axiovert 200M microscope with a ×10 Plan-Neofluar objective (ZEISS) and a Chromyx 4k Pro camera.

### Gut-on-a-chip experiments

Gut-on-chips were assembled according to Feile et al.^36^. In brief, a biochip (BC002, Dynamic42) consisting of an upper and lower channel, which is divided by a porous PET-membrane of 8-µm pore size, was seeded with human umbilical vein endothelial cells and peripheral blood mononuclear cell (PBMC)-derived macrophages in the upper channel and C2BBe1 cells in the lower channel. After the stepwise buildup of the biochips over 4 days, the chips were continuously, circularly perfused via peristaltic pumps at 50 µl min^−1^. After 96 h of pre-perfusion, chips were treated with 100 ng ml^−1^ lipopolysaccharide from *Escherichia* *coli* (Sigma-Aldrich) for 48 h. Then, the biochips were used for specific treatment conditions.

The biochips were exposed to three different supernatants of fibroblast cultures (unstimulated, IF-polarized or IF-polarized and belinostat-treated) on the vascular side (upper channel). For this, chips were exposed to 300 µl of the normal cell culture medium mixed with 200 µl of fibroblast supernatant (500 µl total volume) for 24 h, after which they were perfused with 1 × 10^6^ polymorphonuclear cells (PMNs) on the vascular side. PMNs passed through the upper channel once to allow attachment before further cultivation in the chip under constant circular perfusion for an additional 4 h.

PBMC-derived macrophages were derived from peripheral EDTA-blood from healthy donors. PBMCs were isolated using a density gradient (Histopaque, Sigma-Aldrich) and monocytes were purified by cell-specific adhesion to the plastic bottom of a six-well plate. Monocytes were differentiated into macrophages using 10 ng ml^−1^ granulocyte–macrophage colony-stimulating factor and 10 ng ml^−1^ M-CSF for a total of 9 days. PMNs were isolated from the same donors as the PBMC-derived macrophages using a density gradient (PolymorphPrep, Progen). After PMN isolation, cells were counted and diluted to a concentration of 1 × 10^6^ cells per ml in EC-medium. For each condition, 1 ml of this solution was used to perfuse PMNs through the vascular channel of the biochips. After 24 + 4 h, chips were disconnected from the pump, and both channels were carefully washed with 1 ml of fresh PBS. Subsequently, the chips were opened by precisely removing the upper bonding foil, and the tissue-covered membrane was transferred into RLT Buffer for RNA isolation using the QIAGEN RNeasy kit. The epithelial part of the chip was transferred into methanol for fixation and was subsequently stained via immunofluorescence according to Feile et al.^36^ using the antibodies mouse anti-CD66b (BioLegend, 305102, 1:100 dilution); goat anti-ZO-1 (Invitrogen, PA5-19090, 1:100 dilution); and rabbit anti-E-cadherin (Cell Signaling, 3195S, 1:100 dilution). IF images were captured using a ZEISS Apotome 2 by acquiring a 20–50 µm *z*-stack, which was projected into a two-dimensional *z*-plane.

### RNA interference

ON-TARGETplusSMARTpool PRDM1(L-009322-00), RELB (L-004767-00), ETV4 (L-004207-00) (Horizon Discovery) were diluted in MEMα (Thermo Fisher Scientific) and incubated for 5 min at room temperature. Lipofectamine RNAiMAX (Thermo Fisher Scientific) was diluted (1:20) in MEMα and incubated for 5 min before being mixed with an equal volume of diluted siRNA. The transfection complex was incubated at room temperature for 15 min and further diluted in MEM to achieve a final siRNA concentration of 16.5 nM per well. Human intestinal fibroblasts (Ccd-18Co) were primed using the in vitro inflammatory fibroblast model and transfected with the transfection mix for 24 h. The cells were further cultured in antibiotic free MEMα with 20% FBS (Thermo Fisher Scientific) for an additional 48 h. RNA was isolated using the RNeasy mini kit (QIAGEN) as per manufacturer’s instructions including a RNase-free DNase treatment (QIAGEN). cDNA was synthesized using the High-Capacity cDNA Reverse Transcription kit (Applied Biosystems) and Target gene expression was measured though qPCR using the Precision FAST master mix (Primer Design). Target-specific TaqMan Gene Expression Assays (PRDM1 (Hs00153357_m1), RELB (Hs00232399_m1), ETV4 (Hs00383361_g1), CXCL5 (Hs01099660_g1), CXCL8 (Hs00174103_m1) and MMP1 (Hs00899658_m1)) were used to test relative target gene expression.

### Neutrophil isolation and activation assay, flow cytometry

Primary human neutrophils were isolated from peripheral venous blood of a healthy donor using a MACSxpress Whole Blood Neutrophil Isolation kit (130-104-434; Miltenyi Biotec) according to the manufacturer’s instructions. Isolated neutrophils were resuspended in autologous donor serum and subsequently seeded in 96-well plates (4591; Corning) using 10^6^ cells and 50 µl per well. Cells were treated with 50 µl serum-free MEMα medium or with supernatants from Ccd18-Co fibroblasts that were either nonstimulated and DMSO-treated, cytokine-mix-stimulated and DMSO-treated, or cytokine-mix-stimulated and belinostat-treated. Cells were incubated for 4 h at 37 °C in a humidified tissue-culture incubator (5% CO_2_) and stained for flow cytometric analysis with a fixable Live/Dead dye (1:1,000 dilution in PBS) and an antibody panel targeting CD45 (304024; BioLegend), CD3 (317318; BioLegend), CD16 (360732; BioLegend), CD14 (565283; BD Biosciences), CD66b (305122; BioLegend), CXCR2 (320706; BioLegend) and CXCR1 (320605; BioLegend; all 1:200 dilution in PBS, 0.1% w/v BSA and 1 mM EDTA). Cells were fixed using Fixation Buffer (420801; BioLegend) and analyzed on a BD LSRFortessa flow cytometer.

### Histopathology scoring and analysis

Histopathologic scores of the presence or absence of pathologic features were prepared by a certified GI histopathologist. The free-text description of this scoring was converted in binary presence or absence of established features and analyzed.

### ELISA and Luminex

R&D systems ELISA Duo Set or Quantikine kits were used as per manufacturer’s instructions to measure concentrations of human CXCL5 (DY-254, DX000) in cell culture supernatants, collected for 72 h in serum-free medium. Multi-analyte Luminex kits (R&D systems) were custom designed, used as per the manufacturer’s instructions and acquired on a Luminex MAGPIX.

### Reporting summary

Further information on research design is available in the Nature Portfolio Reporting Summary linked to this article.

## Online content

Any methods, additional references, Nature Portfolio reporting summaries, source data, extended data, supplementary information, acknowledgements, peer review information; details of author contributions and competing interests; and statements of data and code availability are available at 10.1038/s41590-026-02617-0.

## Supplementary information

Supplementary Information Supplementary Figs. 1 and 2.

Reporting Summary

Supplementary Table 1 Patient cohorts and profiled tissues. M, male; F, female; C, currenat; N, never; MP, 6-mercaptopurine; AZA, azathioprine; MTX, methotrexate; ADA, adalimumab; UST, ustekinumab; IFX, infliximab; VD, vedolizumab; UPA, upadacitinib; NA, not available.

Supplementary Table 2 Marker transcripts of cell states detected by 10x Xenium profiling.

Supplementary Table 3 Fold enrichment of Xenium cell states within cellular niches. CellCharter results of detected cellular states within niches.

Supplementary Table 4 IL-1β-induced differential gene expression in intestinal stroma. DESeq2 results of IL-1β stimulation over unstimulated condition.

Supplementary Table 5 IL-13-induced differential gene expression in intestinal stroma. DESeq2 results of cytokine stimulation over unstimulated condition.

Supplementary Table 6 IFNα-induced differential gene expression in intestinal stroma. DESeq2 results of cytokine stimulation over unstimulated condition.

Supplementary Table 7 **I**FNγ-induced differential gene expression in intestinal stroma. DESeq2 results of cytokine stimulation over unstimulated condition.

Supplementary Table 8 IL-17A-induced differential gene expression in intestinal stroma. DESeq2 results of cytokine stimulation over unstimulated condition.

Supplementary Table 9 TGF-β1-induced differential gene expression in intestinal stroma. DESeq2 results of cytokine stimulation over unstimulated condition.

Supplementary Table 10 TNF-induced differential gene expression in intestinal stroma. DESeq2 results of cytokine stimulation over unstimulated condition.

Supplementary Table 11 IL-6-induced differential gene expression in intestinal stroma. DESeq2 results of cytokine stimulation over unstimulated condition.

Supplementary Table 12 OSM-induced differential gene expression in intestinal stroma. DESeq2 results of cytokine stimulation over unstimulated condition.

Supplementary Table 13 IL-22-induced differential gene expression in intestinal stroma. DESeq2 results of cytokine stimulation over unstimulated condition.

Supplementary Table 14 IFNα-induced differential open chromatin in intestinal stroma. DESeq2 results of cytokine stimulation over unstimulated condition.

Supplementary Table 15 IL-22-induced differential open chromatin in intestinal stroma. DESeq2 results of cytokine stimulation over unstimulated condition.

Supplementary Table 16 TGF-β1-induced differential open chromatin in intestinal stroma. DESeq2 results of cytokine stimulation over unstimulated condition.

Supplementary Table 17 IL-1β-induced differential open chromatin in intestinal stroma. DESeq2 results of cytokine stimulation over unstimulated condition.

Supplementary Table 18 IL-17A-induced differential open chromatin in intestinal stroma. DESeq2 results of cytokine stimulation over unstimulated condition.

Supplementary Table 19 IL-6-induced differential open chromatin in intestinal stroma. DESeq2 results of cytokine stimulation over unstimulated condition.

Supplementary Table 20 IFNγ-induced differential open chromatin in intestinal stroma. DESeq2 results of cytokine stimulation over unstimulated condition.

Supplementary Table 21 OSM-induced differential open chromatin in intestinal stroma. DESeq2 results of cytokine stimulation over unstimulated condition.

Supplementary Table 22 IL-13-induced differential open chromatin in intestinal stroma. DESeq2 results of cytokine stimulation over unstimulated condition.

Supplementary Table 23 TNF-induced differential open chromatin in intestinal stroma. DESeq2 results of cytokine stimulation over unstimulated condition.

Supplementary Table 24 Marker open chromatin peaks of stromal cell states detected by 10x Chromium scATAC-seq profiling.

Supplementary Table 25 Marker transcripts of stromal cell states detected by 10x Chromium scRNA-seq profiling.

Supplementary Table 26 Transcript factor loadings of cNMF transcriptional programs.

Supplementary Table 27 Open chromatin peak loadings of cNMF epigenetic programs.

Supplementary Table 28 SCENIC+ regulon statistics for TFs and target genes.

Supplementary Table 29 Table of TFs with fold change statistics by fibroblast cell state.

Supplementary Table 30 SCENIC+ cell type specificity scores of eRegulons (RSS).

Supplementary Table 31 Cytokine mix induced transcriptional changes in intestinal stroma. DESeq2 results of cytokine mix stimulation over unstimulated.

Supplementary Table 32 Belinostat induced transcriptional changes in intestinal stroma. DESeq2 results of belinostat + cytokine mix stimulation over cytokine mix stimulation alone.

Supplementary Table 33 GSK-J4 induced transcriptional changes in intestinal stroma. DESeq2 results of GSK-J4 + cytokine mix stimulation over cytokine mix stimulation alone.

Supplementary Table 34 Cytokine mix induced open chromatin changes in intestinal stroma. edgeR results of cytokine mix stimulation over unstimulated condition.

Supplementary Table 35 Belinostat induced open chromatin changes in intestinal stroma. edgeR results of belinostat + cytokine mix stimulation over cytokine mix stimulation alone.

## Data Availability

10x single-cell and Xenium data are available on the Broad Institute Single Cell portal: scRNA-seq (accession code SCP3254); scATAC-seq (accession code SCP3258); and Xenium 5k (accession code SCP3259). All raw and processed sequencing data are available in the Gene Expression Omnibus under accession code GSE307670. All other data are available in the article and supplementary files.

## Code availability

Scripts and notebooks are available on GitHub at https://github.com/Teichlab/crohns-fibroblast-atlas.
