# Supplementary material for: Epigenetic modulation of stromal cell states underpins pathological tissue niches in Crohn’s disease
Source: Nat Immunol. 2026 Aug 12;27(9):1954–68. doi: 10.1038/s41590-026-02617-0 (PMC13506348; doi:10.1038/s41590-026-02617-0)
Supplement: Supplementary file 1 [file 41590_2026_2617_MOESM1_ESM.pdf]

nature immunology

Supplementary information

https://doi.org/10.1038/s41590-026-02617-0

Epigenetic modulation of stromal cell states underpins pathological tissue niches in Crohn's disease

In the format provided by the authors and unedited

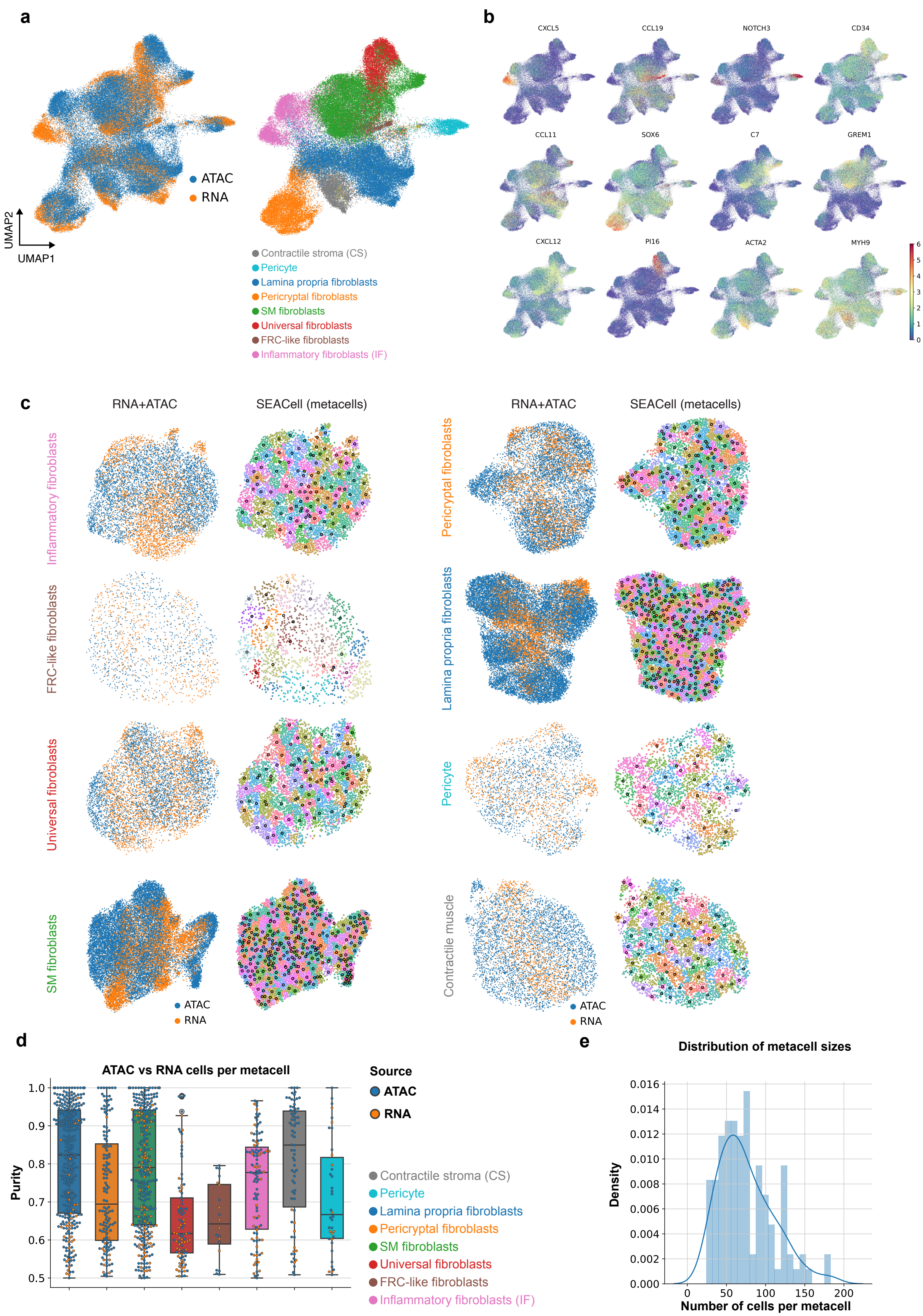
a
ATAC
RNA
UMAP2
UMAP1
Contractile stroma (CS)
Pericyte
Lamina propria fibroblasts
Pericryptal fibroblasts
SM fibroblasts
Universal fibroblasts
FRC-like fibroblasts
Inflammatory fibroblasts (IF)
b
CXCL5
CCL19
NOTCH3
CD34
CCL11
SOX6
C7
GREM1
CXCL12
PI16
ACTA2
MYH9
6
5
4
3
2
1
0
c
RNA+ATAC
SEACell (metacells)
Inflammatory fibroblasts
FRC-like fibroblasts
Universal fibroblasts
SM fibroblasts
Pericryptal fibroblasts
Lamina propria fibroblasts
Pericyte
Contractile muscle
ATAC
RNA
d
ATAC vs RNA cells per metacell
Purity
1.0
0.9
0.8
0.7
0.6
0.5
Source
ATAC
RNA
Contractile stroma (CS)
Pericyte
Lamina propria fibroblasts
Pericryptal fibroblasts
SM fibroblasts
Universal fibroblasts
FRC-like fibroblasts
Inflammatory fibroblasts (IF)
e
Distribution of metacell sizes
Density
0.016
0.014
0.012
0.010
0.008
0.006
0.004
0.002
0.000
0
50
100
150
200
Number of cells per metacell

Supplementary Figure 1. MultiMAP integration and metacell matching of scRNA-seq and scATAC-seq data from intestinal fibroblasts. Annotation was performed first on scRNA-seq data shown in Fig. 3g and the labels then transferred to clusters in the scATAC-seq data shown in Fig 3f. **a,** UMAP of global MultiMAP integration showing integration of cell types and data modalities (ATAC and RNA), validating the label transfer and good mixing of most cell types quantitatively comparable to state-of-the-art integration results in the MulitMAPpaper. **b**, UMAPs showing marker expression. For ATAC data, gene scores are shown, highlighting that differences in IF integration are due to relative differences in expression and chromatin gene scores in CXCL5 and other markers. **c**, UMAPs of subtype-specific MultiMAP integration with SEACell metacell assignments to map cells within fibroblast cel types. **d**, Boxplot showing the mixing by each SEACell metacell for subtype-specific integrations. We used <0.9 purity cutoff to exclude metacells with few matching RNA or ATAC data. **e**, Distribution of cells (ATAC or RNA) per SEACell metacell for inflammatory fibroblast (IF) integration after filtering low-matching (high purity) metacells.

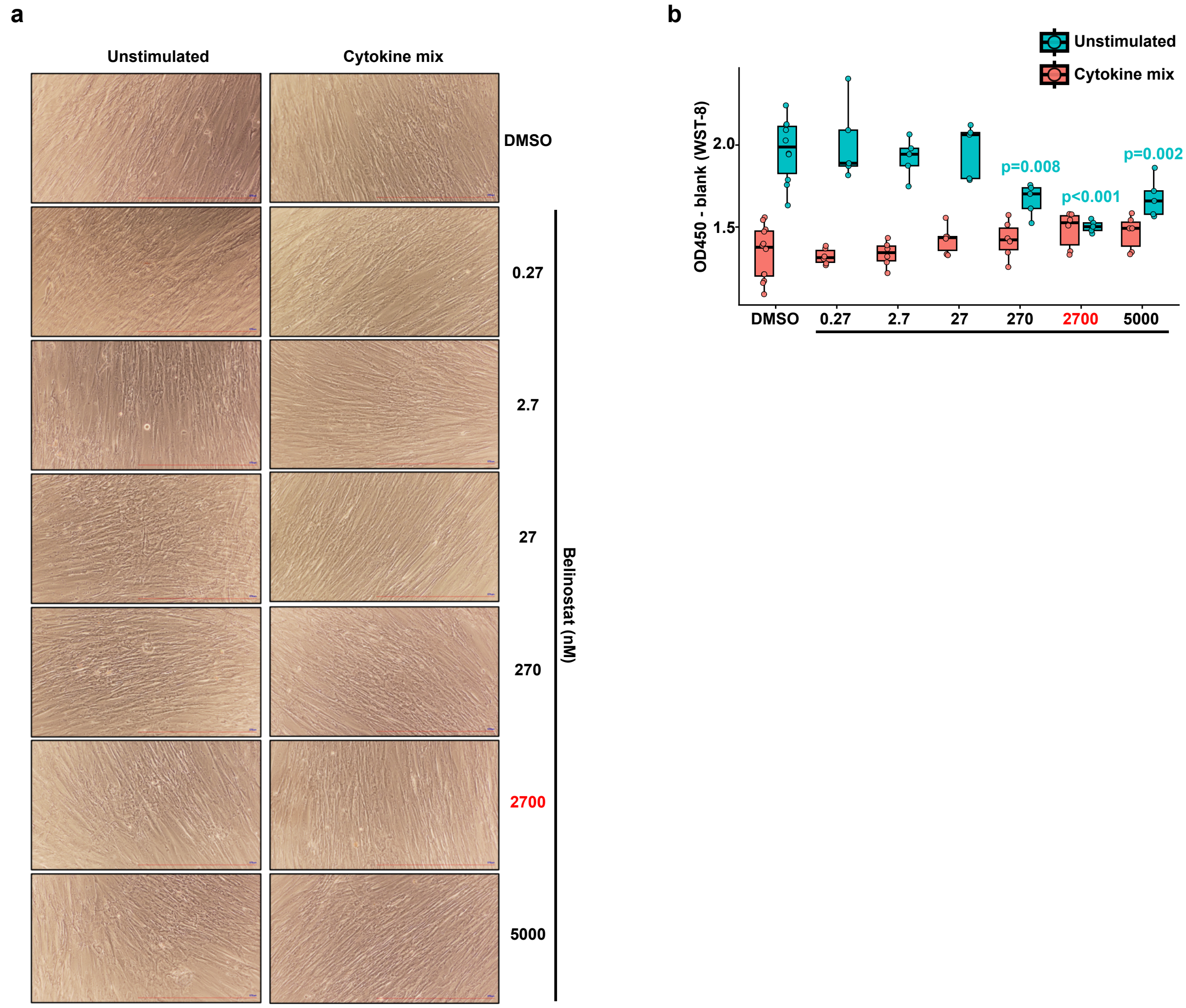

Supplementary Figure 2. Effects of Belinostat on intestinal fibroblasts. a, Representative brightfield images of Ccd18-Co intestinal fibroblast cultures (10x objective), either left unstimulated (left) or subjected to 3 pulses of cytokine mix (right) as depicted in Figure 6a. After cytokine pulses, cultures were subjected to 5 days of Belinostat treatment at different concentrations or DMSO vehicle control. Images were acquired immediately after Belinostat or DMSO treatment, and show comparable cell viability and morphology between all groups. **b,** Measurement of cell metabolic activity after Belinostat or DMSO treatment as in a, using colometric WST-8 assay (n=5-6 replicates per treatment group). Shortly after the 3 cytokine pulses, Ccd18-Co show a higher metabolic activity, which is significantly (post-hoc ANOVA corrected for multiple testing) reduced with Belinostat at active concentrations (270, 2700 and 5000 nM). 2700nM Belinostat (red) was the concentration used in all assays throughout the study, unless stated otherwise.
