# Supplementary material for: Epigenetic modulation of stromal cell states underpins pathological tissue niches in Crohn’s disease
Source: Nat Immunol. 2026 Aug 12;27(9):1954–68. doi: 10.1038/s41590-026-02617-0 (PMC13506348; doi:10.1038/s41590-026-02617-0)
Supplement: Supplementary file 3 [file 41590_2026_2617_MOESM3_ESM.pdf]

| | | | At tissue collection | | | | | | | | | | | | | | | Ever before | |
|---|---|---|---|---|---|---|---|---|---|---|---|---|---|---|---|---|---|---|---|
| **Patient #** | **Tissue sample #** | **Profiling/ Use** | **Diagnosis** | **Sampling site** | **Tissue status** | **Age** | **Sex** | **BMI** | **Smoking** | **Disease duration (years)** | **Disease extent** | **Perianal disease** | **Aminosalicylates** | **Immunomodulators** | **Steroids** | **Biologics** | **Washout previous biologics** | **Immunomodulators** | **Biologics** |
| 1 | 1 | Xenium | CD | Ileum | Adjacent | 30 | F | 25.56 | N | 4 | I3 - ileocolonic | Yes | None | None | None | UPA | None | AZA | IFX, ADA, VDZ, UST |
| 1 | 2 | Xenium | CD | Ileum | Affected | 30 | F | 25.56 | N | 4 | I3 - ileocolonic | Yes | None | None | None | UPA | None | AZA | IFX, ADA, VDZ, UST |
| 2 | 3 | Xenium | CD | Ileum | Adjacent | 55 | F | 41.1 | EX | 7 | I1 - ileal | No | None | None | None | ADA | None | AZA | None |
| 2 | 4 | Xenium | CD | Ileum | Affected | 55 | F | 41.1 | EX | 7 | I1 - ileal | No | None | None | None | ADA | None | AZA | None |
| 3 | 5 | Xenium | CD | Ileum | Adjacent | 32 | F | 19.7 | C | 18 | I3 - ileocolonic | Yes | None | None | None | UPA | None | AZA | ADA, IFX, UST, UPA |
| 3 | 6 | Xenium | CD | Ileum | Affected | 32 | F | 19.7 | C | 18 | I3 - ileocolonic | Yes | None | None | None | UPA | None | AZA | ADA, IFX, UST, UPA |
| 4 | 7 | Xenium | CD | Ileum | Adjacent | 60 | F | 29.2 | EX | 9 | I1 - ileal | No | None | None | None | VDZ | None | AZA | ADA, VDZ, UST |
| 4 | 8 | Xenium | CD | Ileum | Affected | 60 | F | 29.2 | EX | 9 | I1 - ileal | No | None | None | None | VDZ | None | AZA | ADA, VDZ, UST |
| 5 | 9 | Xenium | CD | Ileum | Adjacent | 21 | M | 24.4 | N | 6 | I1 - ileal | No | None | None | None | UST | None | None | UST |
| 5 | 10 | Xenium | CD | Ileum | Affected | 21 | M | 24.4 | N | 6 | I1 - ileal | No | None | None | None | UST | None | None | UST |
| 6 | 11 | Xenium | CD | Ileum | Adjacent | 19 | F | 25.0 | N | 10 | I3 - ileocolonic | No | None | None | None | None | None | AZA | IFX, ADA, UST |
| 6 | 12 | Xenium | CD | Ileum | Affected | 19 | F | 25.0 | N | 10 | I3 - ileocolonic | No | None | None | None | None | None | AZA | IFX, ADA, UST |
| 7 | 13 | Xenium | CD | Colon | Adjacent | 60 | F | 19.4 | N | 25 | I2 – colonic | No | NA | NA | NA | NA | NA | NA | NA |
| 7 | 14 | Xenium | CD | Colon | Affected | 60 | F | 19.4 | N | 25 | I2 – colonic | No | NA | NA | NA | NA | NA | NA | NA |
| 8 | 15 | Xenium | CD | Ileum | Adjacent | 62 | F | 16.9 | EX | 23 | I3 - ileocolonic | Yes | None | None | None | UST | None | AZA | IFX, ADA, VDZ, UST |
| 8 | 16 | Xenium | CD | Colon | Affected | 62 | F | 16.9 | EX | 23 | I3 - ileocolonic | Yes | None | None | None | UST | None | AZA | IFX, ADA, VDZ, UST |

| | | | | | | | | | | | | | | | | | | |
|---|---|---|---|---|---|---|---|---|---|---|---|---|---|---|---|---|---|---|
| 9 | 17 | ScRNAseq, scATACseq | CD | Ileum | Affected | 60 | M | 18.52 | C | 2 | I3 - ileocolonic | No | None | MP | None | ADA | None | None | None |
| 9 | 18 | ScRNAseq, scATACseq | CD | Ileum | Adjacent | 60 | M | 18.52 | C | 2 | I3 - ileocolonic | No | None | MP | None | ADA | None | None | None |
| 10 | 19 | ScRNAseq, scATACseq | CD | Ileum | Affected | 24 | M | 21.46 | N | 6 | I3 - ileocolonic | No | None | None | None | UST | None | None | IFX, ADA, VDZ |
| 10 | 20 | ScRNAseq, scATACseq | CD | Ileum | Adjacent | 24 | M | 21.46 | N | 6 | I3 - ileocolonic | No | None | None | None | UST | None | None | IFX, ADA, VDZ |
| 11 | 21 | ScRNAseq, scATACseq | CD | Colon | Affected | 42 | F | 22.39 | N | 18 | I3 - ileocolonic | No | None | AZA | None | None | None | None | IFX |
| 12 | 22 | ScRNAseq, scATACseq | CD | Ileum | Affected | 67 | M | 28.06 | N | 11 | I3 - ileocolonic | No | None | None | None | IFX | None | AZA, MTX | IFX |
| 12 | 23 | ScRNAseq, scATACseq | CD | Ileum | Adjacent | 67 | M | 28.06 | N | 11 | I3 - ileocolonic | No | None | None | None | IFX | None | AZA, MTX | IFX |
| 13 | 24 | ScRNAseq, scATACseq | CD | Ileum | Affected | 31 | F | 21.07 | EX | 2 | I3 - ileocolonic | No | None | None | None | None | None | AZA | None |
| 13 | 25 | ScRNAseq, scATACseq | CD | Colon | Adjacent | 31 | F | 21.07 | EX | 2 | I3 - ileocolonic | No | None | None | None | None | None | AZA | None |
| 14 | 26 | ScRNAseq, scATACseq | CD | Ileum | Affected | 58 | F | 43.13 | EX | <1 | I1 - ileal | No | None | MTX | None | None | None | None | None |

| | | | | | | | | | | | | | | | | | | | |
|---|---|---|---|---|---|---|---|---|---|---|---|---|---|---|---|---|---|---|---|
| 14 | 27 | ScRNAseq, scATACseq | CD | Colon | Adjacent | 58 | F | 43.13 | EX | <1 | I1 - ileal | No | None | MTX | None | None | None | None | None |
| 15 | 28 | ScRNAseq, scATACseq | CD | Colon | Affected | 42 | F | 17.95 | EX | 18 | I3 - ileocolonic | Yes | None | AZA | None | None | None | None | IFX, ADA |
| 15 | 29 | ScRNAseq, scATACseq | CD | Colon | Adjacent | 42 | F | 17.95 | EX | 18 | I3 - ileocolonic | Yes | None | AZA | None | None | None | None | IFX, ADA |
| 16 | 30 | *In vitro* Stromal expansion | CD | Ileum | Affected | 76 | M | 33.58 | N | NA | I1 - ileal | No | None | None | None | ADA | None | None | None |
| 16 | 31 | *In vitro* Stromal expansion | CD | Ileum | Adjacent | 76 | M | 33.58 | N | NA | I1 - ileal | No | None | None | None | ADA | None | None | None |

**Table S1. Profiled patient cohorts and tissues.** CD = Crohn’s disease; M = male; F = female; C = current; N = never; MP = 6-mercaptopurine; AZA = azathioprine; MTX = methotrexate; ADA = adalimumab; UST = ustekinumab; IFX = infliximab; VDZ = vedolizumab; UPA = upadacitinib; NA = not available.
